# Differential Water Networks Guide Selectivity Optimization of a Cell Active BPTF Inhibitor in Neuroblastoma

**DOI:** 10.1002/anie.4580510

**Published:** 2026-09-08

**Authors:** Kesavan Babu, Chun‐Ju Tsou, Sourav Das, Liying Fan, Anasuya Pal, Molly Sneddon, Timothy R. Stachowski, Priyanka Samanta, Stanley Nithianantham, Caroline Buchholz, Shi Zhang, Xiang Fu, Rahul S. Kathayat, Wenwei Lin, Yongtao Li, Lei Yang, Taosheng Chen, Marcus Fischer, Anang A. Shelat, William C. K. Pomerantz

**Affiliations:** ^1^ Department of Chemical Biology & Therapeutics MS 1000 St. Jude Children's Research Hospital Memphis TN USA; ^2^ Department of Chemistry University of Minnesota Minneapolis Minnesota USA

**Keywords:** bromodomain, neuroblastoma, selective inhibitor, water networks

## Abstract

Bromodomain PHD finger Transcription Factor (BPTF) is an epigenetic regulator implicated in cancer progression. However, despite its oncogenic significance, highly selective and potent inhibitors of the BPTF bromodomain (BRD) with suitable physicochemical properties are lacking. Previously, we reported **BZ1**, a submicromolar BPTF inhibitor that has off‐target activities. Here, we applied comparative structural biology and molecular modeling to design **BZ2**, a regioisomer of **BZ1** with enhanced selectivity for BPTF over other class I BRDs and class‐IV BRDs such as BRD7 and BRD9. Crystal structures and computational analyses reveal that differential engagement of water networks and polar interactions drives this selectivity. Functional studies demonstrate that genetic disruption of the BPTF BRD or treatment with **BZ2** suppresses neuroblastoma (NB) cell growth. With high potency and improved physicochemical properties, **BZ2** provides a valuable tool for probing the biology of BPTF and represents a promising starting point for advancing BPTF‐targeted drug development.

## Graphical abstract

**BZ2** is a second‐generation Bromodomain PHD finger Transcription Factor (BPTF) inhibitor with improved selectivity over Class I and Class IV BRD  off‐targets identified in our previously reported inhibitor, **BZ1**. Structural analyses reveal that water network engagement is key to this selectivity. Functional studies demonstrate that BRD inhibition reduces neuroblastoma growth, highlighting its potential for epigenetic therapy.

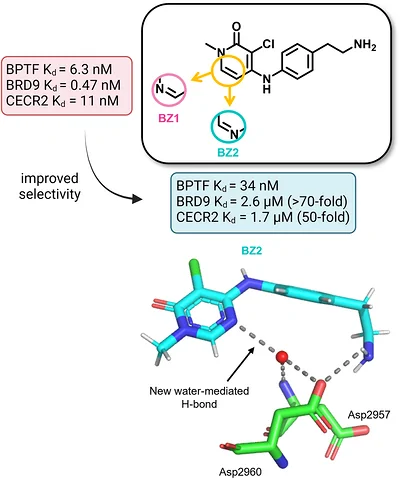

## 1 Introduction

Bromodomains (BRDs) are structurally conserved protein modules that specifically recognize acetylated lysine within proteins. Through these interactions, BRD‐containing proteins engage in transcriptional regulation, nucleosome remodeling, and chromatin modification [1, 2, 3, 4], while their dysregulation is involved in cancer, inflammation, and metabolic diseases [3]. Structural alignments have identified 61 BRDs across 46 different proteins from the human genome. All are comprised of four α‐helices (αZ, αA, αB, and αC) linked by two dynamic loop regions (ZA and BC loops, Figure 1A) and are grouped into eight classes (Figure 1B) [5]. Although class II BRDs, or BRD and extra‐terminal domain (BET) family proteins, are among the most well‐studied, emerging evidence of their role in disease has increased interest in developing therapeutics that target non‐BET BRDs [6, 7].

**FIGURE 1:**
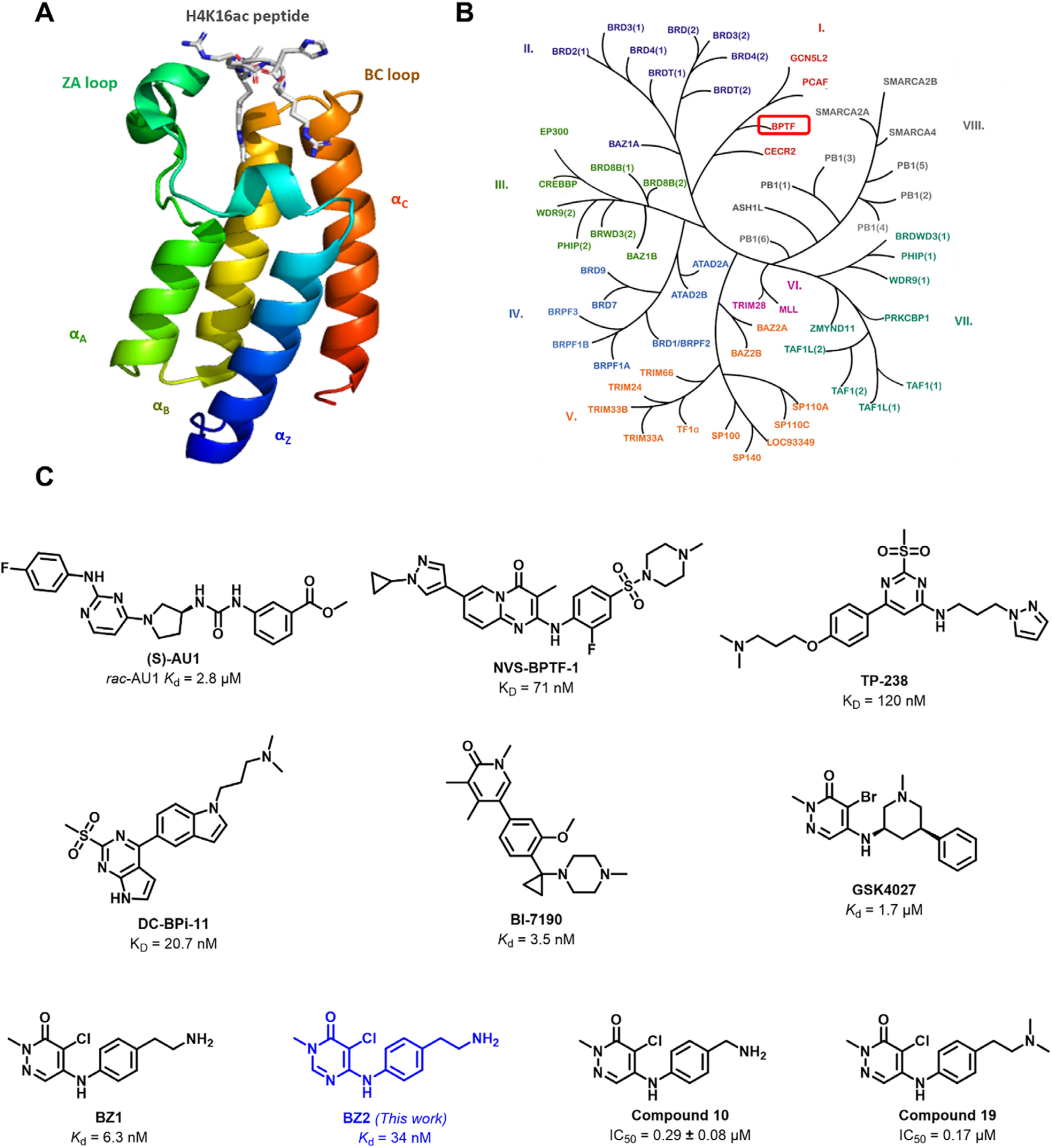
Structure of BPTF BRD and summary of reported inhibitors. (A) Structure of BPTF BRD with bound acetylated histone peptide (PDB ID: 3QZT). (B) BRD phylogenetic tree. (C) Structure of previously reported BPTF BRD inhibitors and **BZ2**.

Bromodomain PHD finger Transcription Factor (BPTF) is a class I BRD and is the largest subunit of the ATP‐dependent nucleosome remodeling factor, NURF. BPTF regulates chromatin structure and, thus, gene expression through its C‐terminal BRD and Plant Homeodomain (PHD) finger domains, which bind to acetylated and methylated histone tails, respectively [8, 9, 10]. BPTF promotes melanocyte stem cell differentiation [11], maintains T‐cell homeostasis [12], and regulates mammary stem cell self‐renewal [13]. Through genetic studies, dysregulation of BPTF is implicated in several adult cancers, including melanoma [14], colorectal [15], bladder [16], lung [17], breast [13], renal cell carcinoma [18], and glioma [19]. Specifically, BPTF has been shown to cooperate with the oncogene MYC [20] by enhancing chromatin accessibility and promoting gene transcription, both through direct interaction with MYC and by upregulating MYC expression. Interestingly, gain of chromosome arm 17q—which contains the BPTF gene locus—occurs frequently in neuroblastoma (NB), which accounts for ∼15% of cancer deaths in children [21, 22]. Notably, a recent analysis in 109 children with NB indicated that high BPTF expression at initial diagnosis was associated with poorer prognosis [23].

Despite being considered druggable, the development of selective, high‐affinity inhibitors for the BPTF BRD has lagged behind that of other BRD‐containing proteins, with limited functional evaluation of inhibition [24]. The first selective BPTF BRD inhibitor, **AU1,** identified in 2015, exhibits a K_d_ of 2.8 µM and shows selectivity over BRD4 (Figure 1C) [25]. **AU1** significantly decreased NB cell colony formation [23] and reduced tumor weight in a murine triple‐negative breast cancer model when combined with doxorubicin [26]. However, **AU1** suffers from poor solubility and stability. **TP‐238**, reported by the Structural Genomics Consortium (SGC), was the first nanomolar BPTF inhibitor but showed 12‐fold stronger binding to CECR2 than BPTF (30 nM vs. 350 nM) [27]. **NVS‐BPTF‐1** (K_d_ = 3 nM), also from SGC, is potent but exhibits off‐target activity within class I BRDs and lacks adequate ADME properties for in vivo use [28]. In 2021, Lu et al. described a **TP‐238** analog, **DC‐BPi‐11** (K_d_ = 20.7 nM), which inhibited the proliferation of MV4‐11 leukemia cells, though its full selectivity profile remains unknown [29]. More recently, Boehringer Ingelheim reported **BI‐7190** (K_d_ = 3.5 nM) with favorable pharmacokinetics, but it has off‐target binding activities with CECR2, BRD9, and CBP [30].

Recently, our group reported a high‐affinity BPTF BRD inhibitor, **BZ1** (K_d_ = 6.3 nM), from modifying **GSK4027**, a PCAF/GCN5L2 inhibitor with modest affinity for BPTF BRD (K_d_ = 1.7 µM) (Table 1) [31]. **BZ1** has acceptable physicochemical properties and more than 350‐fold selectivity over class II BET BRDs, but is limited by significant off‐target activities against the other class I BRD family members and the class IV BRDs, BRD7, and BRD9 [32].

**TABLE 1:** Binding affinities of **BZ1** and **BZ2** to BPTF and relevant off‐targets from the bromoKdELECT assay.

Targets	BZ1 (nM) ^a^	BZ1 selectivity	BZ2 (nM) ^b^	BZ2 selectivity	BZ1/BZ2
BPTF	6.3 ± 1.4	1	34 ± 6	1	5.4
PCAF	12	1.9	375 ± 5	11	32
GCN5L2	44	7	960 ± 340	28	22
CECR2	11	1.7	1750 ± 50	50	150
BRD7	0.76	0.1	2000 ± 0	59	2600
BRD9	0.47 ± 0.4	0.1	2600 ± 300	77	5500

^a^ *K*_d_ values reported in *J. Med. Chem*. **2021**, 64, 18, 13902–13917.

^b^ *K*_d_ values were an average of two replicates, N = 2. Binding isotherms are shown in Figure S2.

Here, we report the design and characterization of **BZ2**, a regioisomer of **BZ1** (Figure 1C). We demonstrate that **BZ2** has enhanced selectivity for BPTF using the bromoKdELECT and AlphaScreen competitive inhibition assays. The compound engages BPTF in cells at submicromolar concentration as measured using a NanoBRET target engagement assay. A complete set of novel x‐ray crystal structures of **BZ1** and **BZ2** each bound to BPTF and BRD9, together with molecular dynamics (MD) simulations, explains the marked improvement of **BZ2's** selectivity. By shifting a nitrogen atom from the pyridazinone ring in **BZ1** to a pyrimidinone ring in **BZ2**, **BZ2** can engage a water network that is specific to BPTF. Finally, we show that either disruption of the BPTF BRD domain using CRISPR base‐editing or treatment with **BZ2** inhibits the growth of SK‐N‐AS NB cells. Taken together, these studies support the potential of **BZ2** as a useful chemical tool to study BPTF biology in cells and as a promising epigenetic therapeutic agent for targeting oncogenic BPTF activity in cancer.

## 2 Results and Discussion

### 2.1 Differential Water Networks Inspired Design for BZ2

To improve the selectivity of **BZ1**, we compared existing structures of BPTF to the other class I members (PCAF, GCN5, CECR2) and class IV BRD7 and BRD9. Unlike the off‐targets, the protein sequence of BPTF has a unique Asp_n_‐x‐x‐Asp_n+3_ pattern in its ZA loop that traps a water molecule (“W1”) via the Asp_n+3_ side chain carboxylate (Asp2960) and the n^th^ residue backbone amide NH (Table S1). This water is consistently observed across x‐ray crystal structures of the **BZ1** analogs, such as **Compound 10** and **19** [32] (PDB: 7RWP, 7RWN) and other ligand‐bound complexes from the PDB (Figure 2A,B). However, it is not present in the structures of BRD7, BRD9, and CECR2, which have a hydrophobic Ile or Tyr residue at n+3; nor in PCAF and GCN5, where the extra carbon atom in the substituted n+3 Glu enables the side chain to directly interact with the backbone amide NH of the Lys residue at the n^th^ position (Figure S1). Docking studies of **BZ1** bound to BPTF (Figure 2C) suggested that relocating the nitrogen from N1 to the C6 position—creating the pyrimidinone analog **BZ2**—would improve selectivity for BPTF over off‐target BRDs. This design was advantageous for three reasons: (1) **BZ2** is less lipophilic than **BZ1** (SLogP 1.37 vs. 1.87); (2) the N1 nitrogen in **BZ2** carries a more negative partial charge than in **BZ1** (OPLS4 charges −0.81 vs. −0.49); and (3) the N2 methyl group in **BZ1** sterically hinders polar interactions at N1. We predicted that **BZ2** would form more hydrogen bonds with bulk water, incurring a higher desolvation penalty. MD simulations confirmed this: N1 in **BZ2** hydrogen‐bonded with bulk water 99% (±1) of the time versus 20% (±2) for **BZ1**. These findings suggest **BZ1** binds BRD7 and BRD9 more strongly due to complementarity with their lipophilic pockets, whereas **BZ2** benefits from stabilization by the W1 water unique to BPTF and is disfavored in the hydrophobic environments of BRD7, BRD9, and CECR2, and would require significant polar rearrangement in PCAF and GCN5 (Figure S1).

**FIGURE 2:**
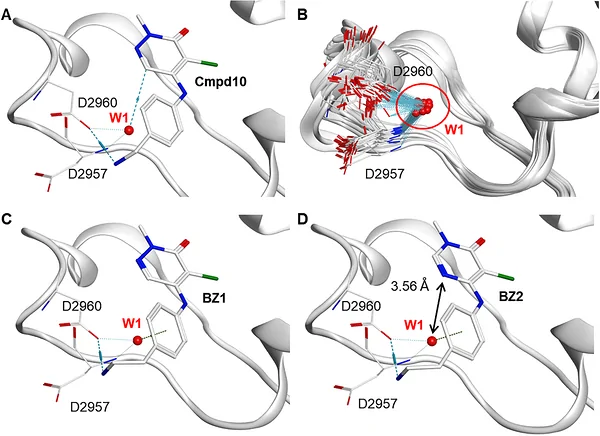
Rationale of **BZ2** design. (A) X‐ray crystal structure of the **BZ1** analog **Compound 10** bound to BPTF (PDB: 7RWP). (B) Alignment of 25 BPTF crystal structures from the PDB showing the conservation of a bound water unique to BPTF. (C) Models of **BZ1** (left) and **BZ2** (right) docked to BPTF with the conserved W1 water. The W1 oxygen atom is 3.56 Å from the nitrogen atom on the pyrimidinone ring of **BZ2**. Dotted lines represent interactions within 4 Å.

### 2.2 Synthesis and Biophysical Characterization of BZ2

To test our structural hypothesis, **BZ2** was synthesized as described in Scheme 1. Using a three‐step synthesis sequence including pyrimidinone methylation, a nucleophilic substitution reaction with a Boc‐protected *p*‐ethylaminoaniline, and subsequent Boc removal led to **BZ2** with a 24% overall yield. Using the commercially available bromoKdELECT assay, we found that despite being 5.4 times less potent against BPTF (K_d_ = 34 nM for **BZ2** vs. 6.3 nM for **BZ1**), **BZ2** was less potent against all off‐targets, resulting in 11 to 77 times higher selectivity for BPTF (Table 1 and Figure S2). We independently confirmed the selectivity trend of **BZ2** for BPTF via an AlphaScreen competition assay using the BPTF, CECR2, and BRD9 BRDs (Table S2), and using AlphaScreen and TR‐FRET for BRD4‐BD1 (Table S2). A negative control compound, **BZ2‐neg**, was synthesized by replacing the secondary amine of **BZ2** with an oxygen atom to disrupt a key hydrogen bond interaction with P2951 (Figure 3 and Scheme 1). As expected, AlphaScreen competition assays of **BZ2**‐**neg** confirmed markedly reduced binding affinity (BPTF IC_50_ = 55 µM).

**SCHEME 1:**
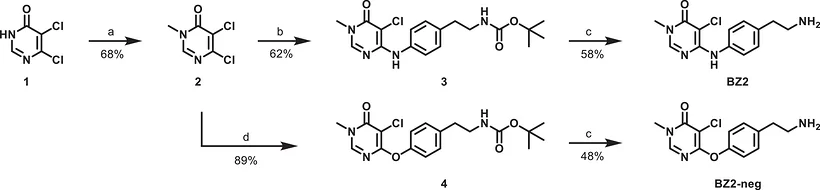
Synthesis of **BZ2**. Reagents and conditions: (a) K_2_CO_3_, methyl iodide, THF, r.t., overnight, (b) tert‐butyl (2‐(4‐amino‐phenyl)‐ethyl)carbamate, DIPEA, DMSO, 120°C, 16 h, (c) trifluoroacetic acid, DCM, r.t., overnight, (d) N‐Boc‐tyramine, K_2_CO_3,_ CH_3_CN, 80°C, 2 h.

**FIGURE 3:**
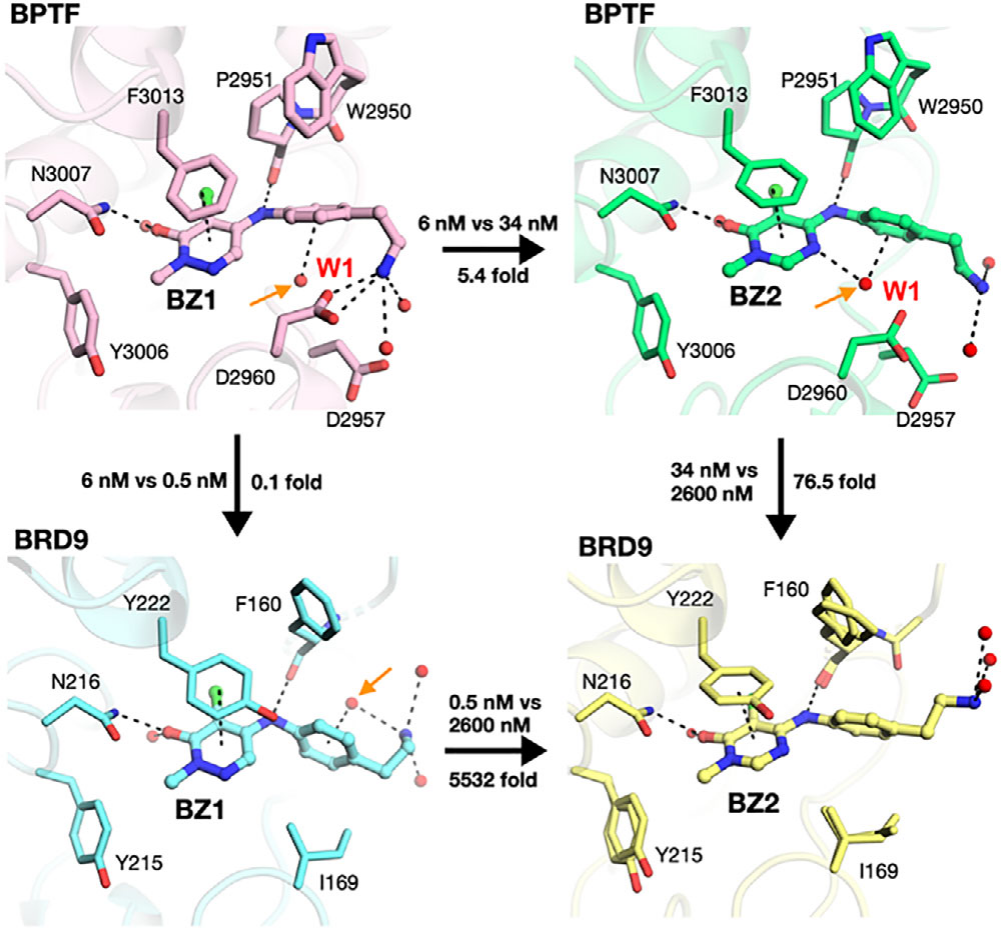
Crystal structures of **BZ1** and **BZ2** with BRD9 and BPTF BRDs enable differential analyses. Four crystal structures of **BZ1** and **BZ2** each bound to BPTF and BRD9 show similarities in the interactions of the pyridazinone and pyrimidinone cores and differences in the placement of the ligand tail. Orange arrows indicate a pi‐stacking water W1 that differs between complexes. Dashed lines represent interactions within 4 Å. Binding affinities and fold‐changes from Table 1 are shown between the structures. Electron density maps are omitted for clarity and shown in Figure S3.

### 2.3 Differential Water Interaction Patterns Drive Changes in Affinity and Selectivity

To experimentally test our computational predictions regarding **BZ1**/**BZ2** selectivity and affinity, we solved crystal structures of BRD proteins that have the highest differences in the affinity and selectivity: BRD9‐**BZ1** (1.05 Å resolution), BRD9‐**BZ2** (1.15 Å), BPTF‐**BZ1** (1.35 Å) and BPTF‐**BZ2** (1.95 Å) (Figures 3 and S3; Table S3). All structures show near‐identical placement of the pyridazinone or pyrimidinone scaffold in the canonical binding pocket (Figure S4) that forms the same interactions as in our preceding analog series (Figure 3) [32]. As predicted by our computational studies, W1 (ID 3285) in BPTF‐**BZ2** sits directly on top of the aromatic ring and hydrogen bonds with the substituted nitrogen (Figure 3B), bridging interactions with Asp2960 and Asp2957 (Figure S5). While W1 is also present in BPTF‐**BZ1**, its geometry is not ideal for engaging Asp2957. The lower normalized B‐factor of W1 in BPTF‐**BZ2** suggests a more ordered interaction with the isosteric nitrogen (Table S4). Engaging W1 may explain the varied position of the aromatic ring and amine tail between BPTF‐**BZ1** and ‐**BZ2**, which then interact with BPTF through different water‐mediated hydrogen‐bonds.

BPTF‐**BZ1** and BRD9‐**BZ1** structures show structural variability of the ring and tail regions (Figures S6 and S7) while most interactions are conserved (Figure 3). This rationalizes the high affinity but poor selectivity of **BZ1**. To investigate why **BZ2** is more selective, we compared BPTF‐**BZ2** and BRD9‐**BZ2**. Again, in BPTF‐**BZ2**, the pi‐stacking W1 sits on top of the aromatic ring and H‐bonds with the substituted nitrogen (Figures 3, 4 and S5C). However, W1 is absent in both BRD9‐**BZ1** and ‐**BZ2** due to the substitution of Asp2960 in BPTF for Ile169 in BRD9 (Figure 3). In contrast, the **BZ1** aromatic ring pi‐stacks with a water (ID 343) on the opposite side, bridging interactions with Gly159, Phe160 and Phe163 (Figures 3 and S6B). These interactions are absent in BRD9‐**BZ2**, as the ring is ∼1.8 Å closer to Phe160. The orientation of the amine tail varies so that in **BZ1** it uses the stacking water to further interact with Gly159, Phe160 and Phe163, while the **BZ2** tail points toward the bulk solvent (Figures 3 and S6C). Taken together, these structural studies provide experimental support for our hypothesis that the presence of the hydrophobic Ile residue at n+3 favors binding to **BZ1** while disrupting binding to **BZ2**, whereas **BZ2** is better accommodated by the W1 water unique to BPTF.

The shifting stacking geometry of W1 between BPTF‐**BZ1**/**BZ2** complexes inspired us to re‐analyze our previous BPTF‐analog series (Figure 4) [32]. Despite different space groups, all structures contain analogous waters with relatively low normalized B‐factors, indicating that the pi‐stacking orientation is stable (Table S4). A BRD9 structure with a different ligand scaffold shows water stacking on the opposite side of the aromatic ring as observed in BRD9‐**BZ1** (Figure 4C, 4I) [33]. Yet in each case, the geometry is less ideal than in BPTF‐**BZ2**, where W1 has the lowest B‐factor, highlighting the stability of the W1‐nitrogen interaction in BPTF‐**BZ2**. Although weak, these lone pair‐pi interactions have been shown to play important roles in protein recognition and stability [34, 35, 36].

**FIGURE 4:**
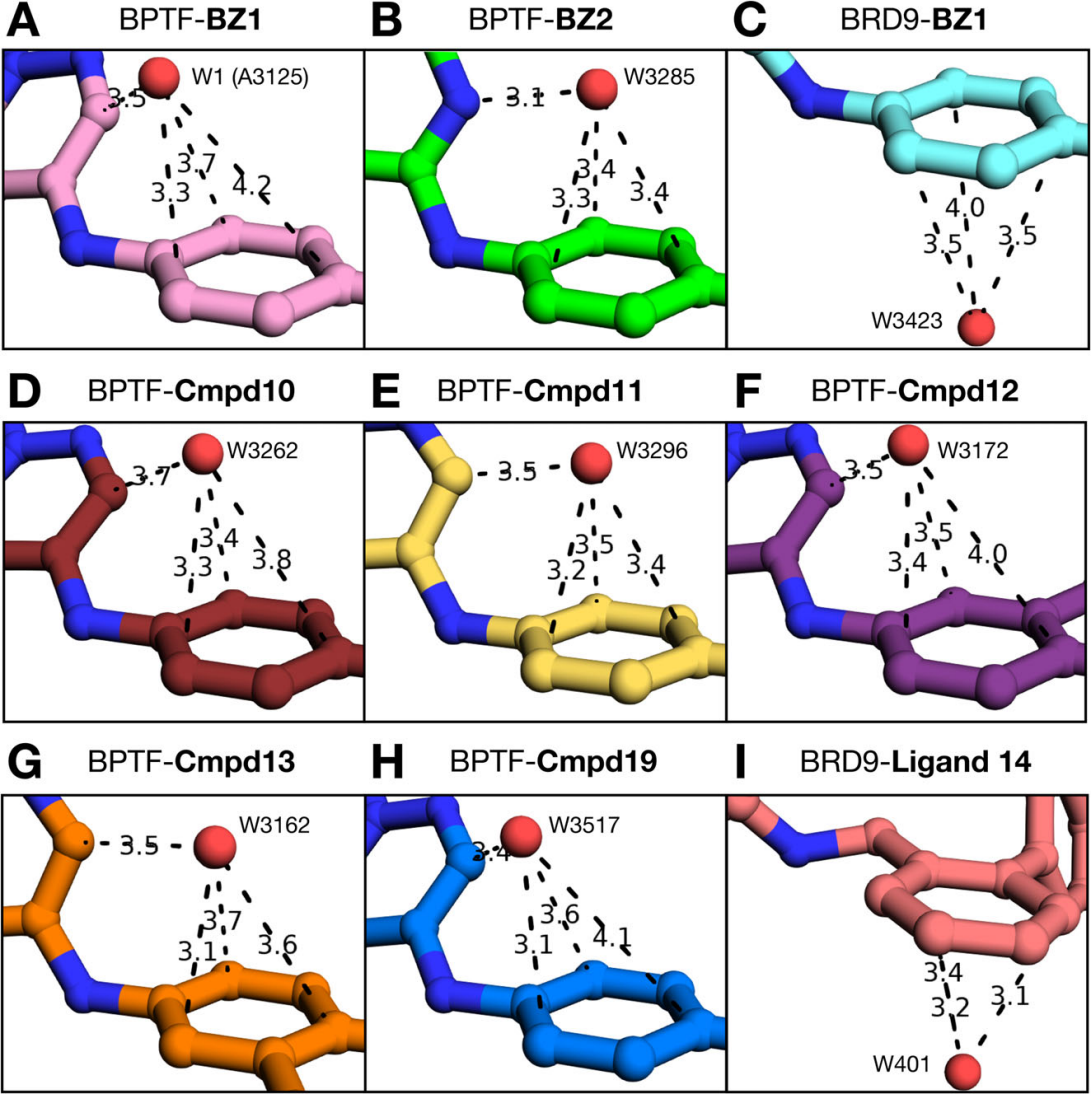
Water‐mediated pi‐interaction in BPTF and BRD9 crystal structures. BPTF (A‐G) and BRD9 (H and I) structures that bound with **BZ1** or **BZ2** or analog compounds [32, 37] show a pi‐stacking W1‐equivalent water (numbered as in respective PDB; Table S4) above the aromatic ring of the inhibitor compounds. Dashed lines are distances in Å, showing the geometry of respective pi‐stacking interactions. BRD9‐**BZ2** is not shown since it does not contain a stacking water.

To further validate water as a key determinant of selectivity, we aligned crystal structures from the PDB for BRD9, CECR2, GCN5L2, PCAF, and BRD7 with Asp2960 of our BPTF‐**BZ2** crystal structure (Figure 5). Waters observed in existing BPTF structures clustered with the W1 water (green) from BPTF‐**BZ2**. In contrast, the W1 location was sterically blocked by Ile196 in BRD9 and BRD7 and by Tyr497 in CECR2, while in GCN5L2, Glu761 occupied the W1 position. Notably, PCAF—the most selective BRD for **BZ2** after BPTF—contained a water cluster and showed variable Glu756 conformations. These findings suggest that among **BZ1** off‐targets, PCAF best accommodates the polar interactions of **BZ2**, providing a structural rationale that mirrors the observed selectivity pattern.

**FIGURE 5:**
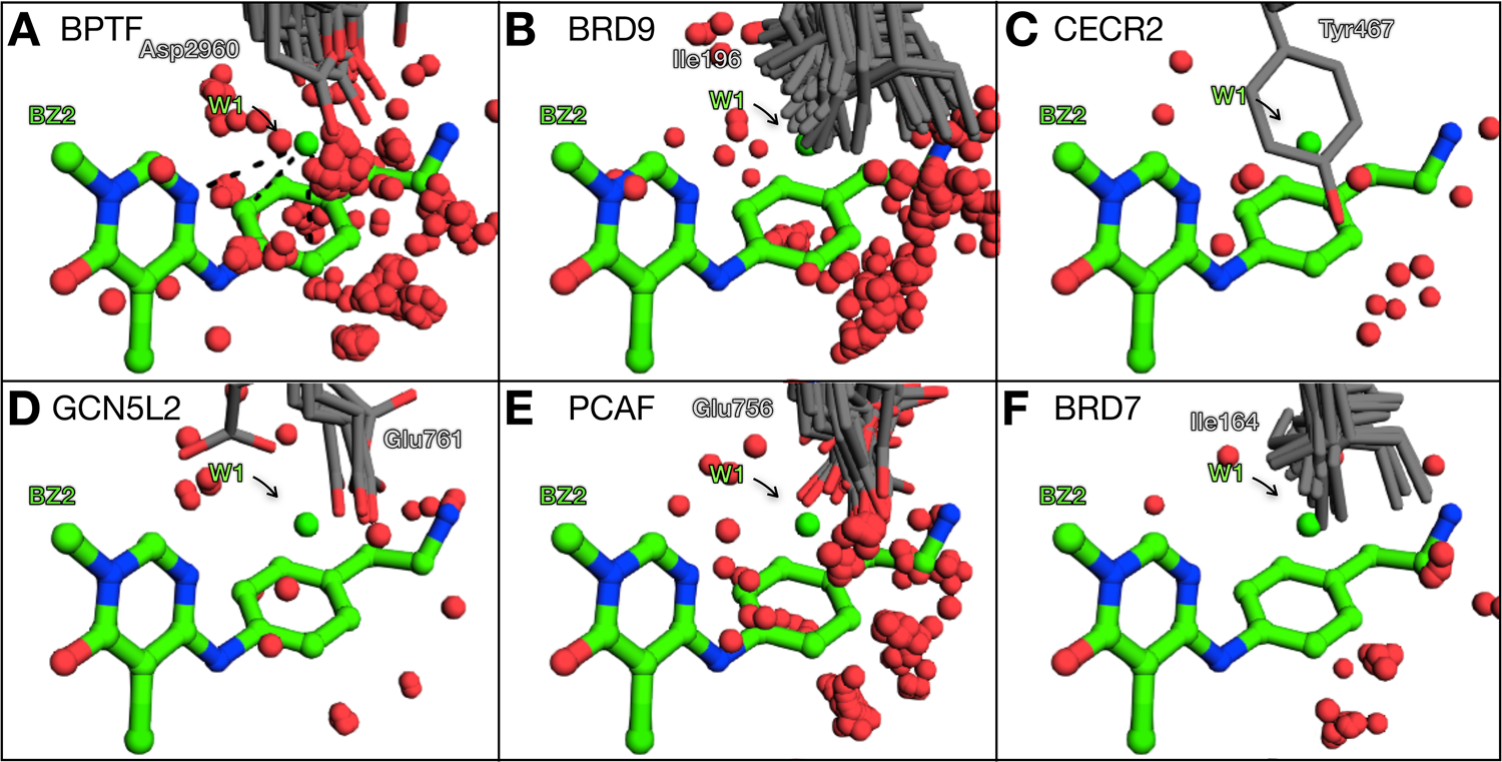
Conservation of waters in binding sites across (A) BPTF, (B) BRD9, (C) CECR2, (D) GCN5L2, (E) PCAF, and (F) BRD7. Structures from the PDB were aligned with residues occupying the same position as Asp2960 from BPTF and are shown in gray. **BZ2** and W1 from the BPTF crystal structure are shown in green for reference.

### 2.4 MD Simulations of BZ1 and BZ2 Bound to BPTF

We conducted a binding free energy analysis to dissect the energetic contributions in binding **BZ1** and **BZ2**. AMBER MM‐GBSA analysis [38] from MD simulations predicted **BZ1** to have a higher affinity (ΔG = ‐6.3 kcal/mol) compared to **BZ2** (Figure 6). Although **BZ2** exhibited a more favorable Coulombic interaction than **BZ1**, likely due to the proximity of the electronegative N1 pyrimidinone nitrogen to the polar region of BPTF, it showed slightly less favorable van der Waals and nonpolar solvation contributions, along with a substantially less favorable polar solvation term. Thus, although the added electrostatic interaction in **BZ2** improved selectivity against off‐targets, its overall effect was to reduce binding affinity for BPTF because of diminished hydrophobic interactions and insufficient compensation for the energetic cost of desolvation.

**FIGURE 6:**
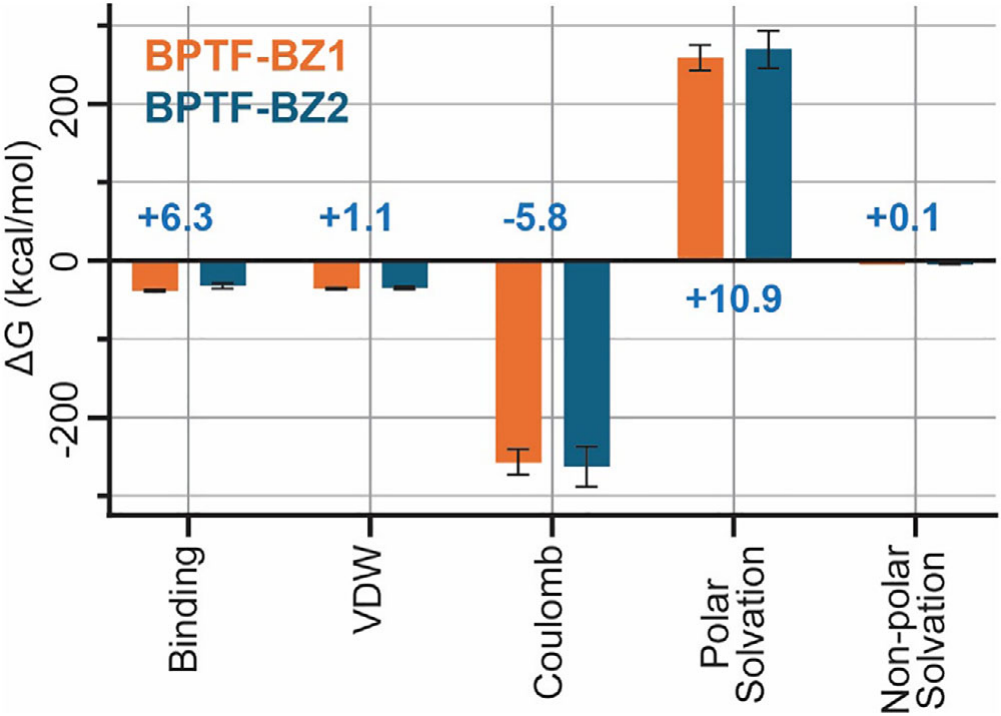
Analysis of ligand‐water interactions in BPTF bound to **BZ1** or **BZ2**. AMBER MM‐GBSA calculations were conducted on the last 50 ns (50 frames) from five independent replicates for each complex. The ΔΔG (**BZ2** – **BZ1**) is shown in blue.

### 2.5 BZ2 Shows Activity in Cellular Models of Neuroblastoma

Although genetic studies have implicated the inhibition of BPTF as a therapeutic target in cancer, it is unclear whether inhibition of the BPTF BRD would be effective. Using reconstituted nucleosomes, Allis et al. showed that the PHD‐disabling point mutation Trp2891Glu more severely impaired BPTF binding compared to the BRD‐disabling point mutation Phe3013Ala [10]. Using an artificial PHD‐BRD construct that was limited to the C‐terminus of BPTF, they also found that introducing either mutation in cells was sufficient to impair binding to chromatin at certain genomic loci. To address a potential discrepancy of using a truncated construct, we applied CRISPR/Cas9 gene editing to SK‐N‐AS NB cells, and isolated stable clones with BPTF knocked out (KO) or mutated with the PHD (Trp2891Glu) or BRD (Phe3013Ala) point mutations. We then pooled SK‐N‐AS BPTF wild‐type (WT), KO, Trp2891Glu, or Phe3013Ala cells and conducted a CelFi cellular fitness assay, whereby the relative population of each cell type was monitored over 21 days [39]. Compared to WT cells, BPTF KO and domain‐edited cells showed diminished fitness at less than 1% of the total population at the end of the experiment (Figure 7A). These data suggest that inhibiting the BPTF BRD would be sufficient to impair growth in SK‐N‐AS cells.

**FIGURE 7:**
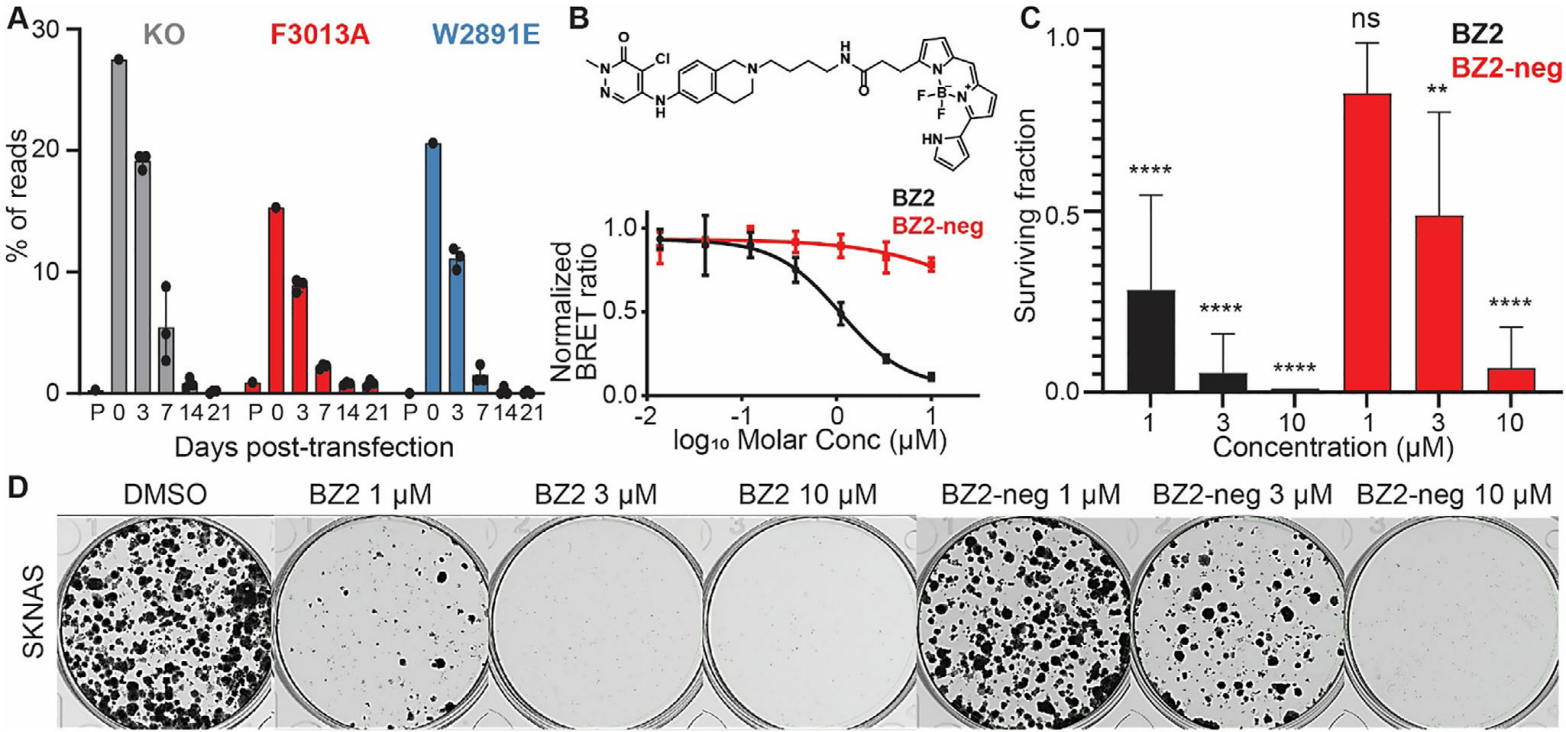
Assessing the impact of BPTF BRD inhibition in NB. (A) Fitness assay of SK‐N‐AS cells that are wild‐type (WT), BPTF knock out (KO), or edited with PHD (Trp2891Glu) domain or BRD (Phe3013Ala) point mutations. (B) NanoBRET in‐cell target engagement assay probe (top) and dose‐response results (bottom) for **BZ2** and **BZ2‐neg** in HEK293T cells. (C) Colony‐forming assay results for SK‐N‐AS cells treated with **BZ2** and **BZ2‐neg** for 14 days. (D) Representative images from the experiment summarized in (C).

To confirm the engagement of **BZ2** with the BPTF BRD in cells, we developed a NanoBRET assay based on the Bioluminescence Resonance Energy Transfer (BRET) between NanoLuc Luciferase fused to BPTF and a cell‐permeable fluorescent tracer that binds reversibly to the BPTF BRD based on our pyrimidinone analogs (Figure 7B). In HEK293T cells, the tracer was blocked by **BZ2** (EC_50_ ∼ 352 nM [279 to 441 nM]), albeit at concentrations higher than its K_d_. Subsequent permeability studies showed that BZ2 exhibited moderate apparent permeability in Caco‐2 cells, was subject to direction‐dependent transport in MDCKII cells, and was identified as likely an MDR1 substrate, suggesting that transporter‐mediated efflux contributes to reduced cellular activity relative to NanoBRET target engagement (Table S5). The negative control compound, **BZ2‐neg**, was inactive as expected (Figure 7B). Finally, we tested **BZ2** and **BZ2‐neg** in a colony‐forming assay using SK‐N‐AS cells and found that **BZ2** significantly inhibited tumor cell growth at concentrations ≥1 µM, whereas **BZ2‐neg** showed comparable efficacy at higher concentrations (Figure 7C,D). While modest, the difference in potency between **BZ2** and its negative control suggests that the cytotoxicity of **BZ2** in NB cells is, in part, dependent on BPTF, but also motivates future efforts to improve cellular potency. Together, these findings show that BPTF BRD inhibition by **BZ2** is sufficient to inhibit SK‐N‐AS cell growth, and suggest BPTF is a promising therapeutic target for NB.

## 3 Conclusion

We report the design and characterization of **BZ2**, a second‐generation inhibitor of the BPTF BRD, which demonstrates greater selectivity for BPTF over other BRDs compared to its predecessor, **BZ1**. The design of **BZ2** was inspired by a unique water molecule (W1) observed in multiple BPTF BRD crystal structures, but absent in BRD7, BRD9, and other class I BRDs. Docking studies and MD simulations predicted that the N1 nitrogen of **BZ2's** pyrimidinone ring would form a hydrogen bond with this water molecule. To test this hypothesis, we synthesized **BZ2** and evaluated its selectivity using two biophysical competitive inhibition assays. These assays showed that **BZ2** is 11–77 times more selective for BPTF over off‐target BRDs. We also solved a systematic set of x‐ray crystal structures for both **BZ1** and **BZ2** bound to BPTF or BRD9. While all complexes displayed similar binding modes, in the BPTF‐**BZ2** structure, W1 was positioned directly above the ligand's aromatic ring, forming a hydrogen bond with the pyrimidinone nitrogen and bridging to neighboring aspartate residues. In contrast, although W1 was present in BPTF‐**BZ1**, its geometry was less ordered and no consistent interaction pattern was observed. The pi‐stacking water seen in BPTF‐**BZ2** was absent in both BRD9 structures, where an isoleucine (Ile169) replaces the aspartate (Asp2960) found in BPTF. Notably, a water molecule on the opposite side of the **BZ1** aromatic ring in BRD9 is missing in BRD9‐**BZ2**, which helps explain BRD9's preference for binding **BZ1** over **BZ2**. Additional BRD crystal structures aligned with BPTF‐**BZ2** revealed that PCAF, the next most selective BRD for **BZ2** after BPTF, contains a water cluster that can support polar interactions with **BZ2**, consistent with our selectivity data.

Although **BZ2** exhibited improved selectivity, its potency against BPTF (K_d_ = 34 nM) was slightly lower than that of **BZ1** (K_d_ = 6.3 nM). MD simulation‐based binding free energy analysis indicated that the increased Coulombic interactions—mainly due to hydrogen bonding with water—did not fully compensate for the loss of favorable lipophilic interactions and the higher desolvation penalty. This investigation highlights the challenge of rationally designing small molecules to target bound water molecules [40, 41]. Furthermore, targeting a unique structured water molecule within the BPTF BRD binding site further illustrates a successful design strategy for improving selectivity among structurally related BRDs. Similar approaches that exploit structured water networks have enabled the development of selective inhibitors for the class II BET BRDs, including BRD4 [42], the engineering of BET bromodomain 1 versus bromodomain 2 selectivity [43, 44], and the tuning of selectivity for Class I, IV, and VII BRDs, including CECR2, BRD9, and TAF1(2), respectively [45]. Together, these findings underscore the significant potential of targeting structured water networks to achieve BRD selectivity.

Finally, to determine if BPTF BRD inhibition alone is sufficient to suppress cancer cell growth, we compared the viability of NB cells with BPTF knockout or with either the PHD or BRD domain disabled. All three edited cell models showed reduced viability compared to the parental cell line, indicating that BRD inhibition alone impairs NB cell growth. We further confirmed that **BZ2** engages its target in cells at high nanomolar concentrations, while the negative control compound (**BZ2‐neg**) was inactive. Importantly, **BZ2** inhibited the growth of SK‐N‐AS cells, whereas **BZ2‐neg** was less active. These results establish **BZ2** as a valuable tool for studying BPTF biology and support its further development as an epigenetic therapy targeting oncogenic BPTF activity in NB and other BPTF‐associated cancers.

## Author Contributions

K.B. and S.N. performed x‐ray crystallography; T.R.S. performed crystallographic refinement and comparative structural analysis; C.T. synthesized the compounds and conducted biophysical experiments; S.Z. performed MD simulations at 300 K; P.S. and S.D. performed MD simulations and molecular docking studies; R.S.K. contributed to optimizing the NanoBRET assay M.S. synthesized the NanoBRET tracer and performed the NanoBRET assay; X.F. and LY performed and oversaw the cell permeability assays; L.F., L.Y., and A.P. conducted neuroblastoma experiments; C.B. synthesized and characterized compounds 10 and 19; W.L., Y.L., and T.C. performed the TR‐FRET studies; M.F., A.S., and W.C.K.P. oversaw the experiments and the interpretation of the data. C.T. and W.C.K.P. wrote the initial draft.

## Conflicts of Interest

The authors declare no conflicts of interest.

## Supporting information



The authors have cited additional references within the Supporting Information.**Supporting File**: anie74704‐sup‐0001‐SuppMat.pdf.

## Data Availability

The data that support the findings of this study are openly available in pdb at https://www.rcsb.org/, reference number PDBID: 8F6G 9ZUK 9ZU3 9ZU4.
