# Supplementary material for: Differential Water Networks Guide Selectivity Optimization of a Cell Active BPTF Inhibitor in Neuroblastoma
Source: Angew Chem Int Ed Engl. 2026 Sep 8;65(44):e4580510. doi: 10.1002/anie.4580510 (PMC13560301; doi:10.1002/anie.4580510)
Supplement: Supplementary file 1 [file ANIE-65-e4580510-s001.pdf]

Differential Water Networks Guide Selectivity Optimization of a Cell Active BPTF Inhibitor in Neuroblastoma

Kesavan Babu#[a], Chun-Ju Tsou#[b], Sourav Das#[a], Liying Fan[a], Anasuya Pal[a], Molly Sneddon[b], Timothy R. Stachowski[a], Priyanka Samanta[a], Stanley Nithianantham[a], Caroline Buchholz[b], Shi Zhang[a], Xiang Fu[a], Rahul S. Kathayat[a], Wenwei Lin[a], Yongtao Li[a], Lei Yang[a], Taosheng Chen[a], Marcus Fischer*[a], Anang A. Shelat*[a], William C. K. Pomerantz*[b]

Supporting Information

Table of Contents

Computational and cell viability assay methods 1

Experimental Section - Chemistry 2

Protein purification and crystallization 7

X-ray diffraction data collection and structure refinement 7

Figure S1. Quantification of polar (red) and lipophilic (green) surface areas in the vicinity of BZ1 docked to BPTF and off-targets. 8

Figure S2. DiscoverX BROMOscan titrations of BZ2 9

Figure S3. Electron density for ligand and stacking waters: 10

Figure S4. Structural superposition of BPTF and BRD9 bound BZ1 and BZ2 11

Figure S5. Comparison of BZ1 and BZ2 bound BPTF structures. 12

Figure S6. Comparison of BRD9-BZ1 and BRD9-BZ2 structures. 13

Figure S7. Comparison of BZ1 in the context of BPTF vs BRD9, and BZ2 in the context of BPTF vs BRD9. 14

Table S1. The Asp_n-x-x-Asp_{n+3} sequence pattern is unique to BPTF 15

General Procedure for the AlphaScreen Assay 16

Table S2. Binding affinities of BZ1 and BZ2 to BPTF and other BRD-containing proteins 17

Table S3. Crystallographic data processing and refinement statistics 18

Table S4. Pi-stacking water stability based on normalized B-factor. 19

Table S5. Cell permeability studies of BZ2. 19

TR-FRET probe synthesis 19

Figures S8-11 NMR spectra, HRMS spectrum and HPLC chromatogram of TR-FRET probe.....21

TR-FRET protocol.. 24

Computational and cell viability assay methods

Molecular dynamics simulations. The BZ1 and BZ2 protein-ligand crystal structures were simulated using AMBERTools26[1] and AMBER26[2] (pmemd.cuda). Ligand parameters were generated with Antechamber using the GAFF2 force field[3] and AM1-BCC partial charges[4], with a net +1 ligand protonation state, and missing bonded parameters were assigned with parmchk2. Protein and complex topologies were built in tleap using the ff14SB force field for the protein[5] and the TIP3P water model[6, 7]. Each complex was solvated in a TIP3P water box with a 15 Å buffer and neutralized with Na^+/Cl^- ions to a final concentration of ~150 mM. Each system was energy-minimized in two stages and then heated from 0 to 300 K in two stages (5 ps at constant volume to 100 K, followed by 100 ps at constant pressure to 300 K) under harmonic positional restraints (5 kcal/mol/Å²) on all non-water, non-ion atoms. Systems were further equilibrated for 1 ns with restraints limited to the ligand and protein backbone atoms (Cα, C, N), followed by 1 ns with restraints limited to Cα atoms only. Production dynamics were run for 500 ns at 300 K and 1 atm (NPT) using a Langevin thermostat, a hydrogen-mass-repartitioned (HMR) topology[8], and SHAKE-constrained bonds to hydrogen, with long-range electrostatics treated by particle mesh Ewald (PME)[9] and a 10 Å real-space cutoff. Five independent 500 ns replicates were performed per complex (BZ1 and BZ2), each with an independently randomized initial velocity seed. Snapshots for MM-GBSA calculations were extracted from each production trajectory at 1 ns intervals.

MM-GBSA calculations. Binding free energies for the AMBER trajectories were calculated using the Molecular Mechanics Generalized Born/Surface Area (MM-GBSA) method implemented in MMPBSA.py[10] within AMBERTools26, applied to the final 50 ns of each 500 ns production trajectory using a single-trajectory protocol. The polar contribution to solvation (ΔG_{GB}) was calculated using the Generalized Born model (igb = 5)[11]. The nonpolar contribution (ΔG_{SA}) was calculated from the LCPO solvent-accessible surface area, with a 0.15 M salt concentration used in both. Because the bonded terms (bond, angle, dihedral) cancel to ~0 in the complex − receptor − ligand difference (no covalent bonds are broken or formed), the individual energy components extracted were the electrostatic/Coulomb energy (ΔE_{ele}), van der Waals energy (ΔE_{vdW}), polar solvation energy (ΔG_{GB}), and nonpolar solvation energy (ΔG_{SA}).[1–11]

Cell viability assay. SK-N-AS cells were cultured as reported[12] and plated in 6-well plates, 3000 cells per well in 2ml media. After 24 hrs of cell plating, the compounds were added, and the plates were incubated at 37'C. The plates were incubated for at least 3 weeks till individual colonies were observed in the wells. After removal of the media from the wells, the colonies were fixed with cold 4% paraformaldehyde (PFA) for 15 mins. After aspirating the PFA, 0.25% crystal violet dye was used to stain the colonies for 20 mins. The plates were carefully washed with water, dried and imaged in the colony counter. A bar graph was generated after colony counting under each treatment. The experiment was repeated n=3 and the graph plotted to show SD (standard deviation).

Experimental Section - Chemistry

Materials and Methods

All commercially available reagents were used without further purification. Flash column chromatography was performed on a Teledyne-Isco Rf-plus CombiFlash instrument with RediSep columns. NMR spectra were collected on a Bruker Avance III AX-400 spectrometer or a Bruker Avance III HD-500 spectrometer equipped with a Prodigy TCI cryoprobe. Chemical shifts (δ) are reported in parts per million (ppm) and referenced to residual solvent signals for chloroform-*d* (^{1}H δ 7.26 ppm) and methanol-d_4 (^{1}H δ 3.31 ppm, ^{13}C δ 49.0 ppm). Coupling constants (*J*) are in Hz. Splitting patterns are reported as s (singlet), d (doublet), t (triplet), q (quartet), and m (multiplet). High-resolution ESI-MS spectra were recorded on a Sciex X500R QTOF system.

5,6-dichloro-3-methylpyrimidin-4(3H)-one (2)

5,6-dichloropyrimidin-4(3*H*)-one (445 mg, 2.70 mmol, 1 equiv), methyl iodide (460 mg, 3.24 mmol, 1.2 equiv), and potassium carbonate (447 mg, 3.23 mmol, 1.2 equiv) were added to THF (2.7 mL). The reaction mixture was stirred at room temperature overnight and was washed with a 10% sodium thiosulfate solution. The resultant mixture was extracted with ethyl acetate (3 × 20 mL), and the filtrate was concentrated in vacuo, yielding 5,6-dichloro-3-methylpyrimidin-4(3*H*)-one (72%). ^{1}H NMR (500 MHz, $CDCl_3$) δ 7.98 (s, 1H), 3.62 – 3.51 (m, 3H); ^{13}C NMR (126 MHz, $CDCl_3$) δ 157.81, 154.66, 147.43, 121.31, 35.27; HRMS (ESI-TOF) calculated for $C_5H_4Cl_2N_2O$ [M + H]$^+$ 178.97734, observed 178.9796.

6-((4-(2-aminoethyl)phenyl)amino)-5-chloro-3-methylpyrimidin-4(3*H*)-one (BZ2)

A mixture of **2** (360 mg, 2.01 mmol, 1 equiv), *tert*-butyl (2-(4-amino-phenyl)-ethyl)carbamate (427 mg, 1.81 mmol, 0.9 equiv), and N,N-diisopropylethylamine (518 mg, 4.02 mmol, 2.0 equiv) was dissolved in DMSO (600 µL) and heated at 120 °C for 16 h. The reaction mixture was extracted with ethyl acetate, washed with 1 M sodium bicarbonate solution (3 × 20 mL), and then with brine (20 mL). The organic layer was then dried with magnesium sulfate, filtered, concentrated in vacuo, and purified by flash column chromatography to yield **3** (644 mg, 84.8% yield). Both 5- and 6-positional isomers were obtained, with the desired compound being the major product. To remove the Boc protecting group, product **3** was stirred in DCM (4 mL) at room temperature, and trifluoroacetic acid (10.0 equiv) was added to the mixture. The reaction was stirred at room temperature overnight, then extracted into DCM, and treated with 1 M NaOH to bring the pH to 10. The DCM layer was then dried over magnesium sulfate, filtered, and concentrated in vacuo to afford the product. ^{1}H NMR (500 MHz, methanol-d_4) δ 8.09 (s, 1 H), 7.44 (d, J = 8.5 Hz, 2H), 7.24 (d, J = 8.5 Hz, 2H), 3.51 (s, 3 H), 3.09 (t, J = 7.2 Hz, 2 H), 2.90 (t, J = 7.7 Hz, 2H); ^{13}C NMR (126 MHz, methanol-d_4) δ 160.6, 157.5, 150.3, 138.7, 135.0, 130.1, 125.1, 97.0, 42.6, 35.6, 34.8; HRMS (ESI-TOF) calculated for $C_{13}H_{15}ClN_4O$ [M + H]$^+$ 279.1007, observed 279.1003.

6-((4-(2-aminoethyl)phenyl)amino)-5-chloro-3-methylpyrimidin-4(3*H*)-one (BZ2-neg)

A mixture of **2** (130 mg, 0.73 mmol, 1 equiv), *tert*-butyl (4-hydroxyphenethyl)carbamate (172 mg, 0.73 mmol, 1 equiv), and potassium carbonate (110 mg, 0.80 mmol, 1.1 equiv) was dissolved in acetonitrile (1.5 mL) and heated at 80 °C for 2 h. The reaction mixture was extracted with ethyl acetate, washed with water (2 × 10 mL), and then with brine (10 mL). The organic layer was then dried with magnesium sulfate, filtered, concentrated in vacuo, and purified by flash column chromatography to yield **4** (160 mg, 57% yield). Both 5- and 6-positional isomers were obtained, with the desired compound being the major product. To remove the Boc protecting group, product **4** (130 mg, 0.73 mmol, 1 equiv) was stirred in 4M HCl in dioxane (0.53 mL, 2.11 mmol, 5 equiv) at room temperature overnight. The mixture was treated with 1 M NaOH to bring the pH to 10, then dried over magnesium sulfate, and purified by flash column chromatography to afford the product. ^{1}H NMR (500 MHz, MeOD) δ 8.16 (s, 1H), 7.34 (d, *J* = 8.5 Hz, 2H), 7.13 – 7.08 (m, 2H), 3.54 (s,

3H), 3.21 (dd, *J* = 8.6, 6.8 Hz, 2H), 3.01 (dd, *J* = 8.6, 6.9 Hz, 2H); ^{13}C NMR (126 MHz, MeOD) δ 164.79, 161.90, 153.32, 150.55, 135.43, 131.06, 122.98, 103.62, 41.92, 35.29, 33.91; HRMS (ESI-TOF) calculated for $C_{13}H_{14}ClN_3O_2$ [M + H]$^+$ 280.0847, observed 280.0852.

NMR spectra

2, ^{1}H NMR (500 MHz, $CDCl_3$)

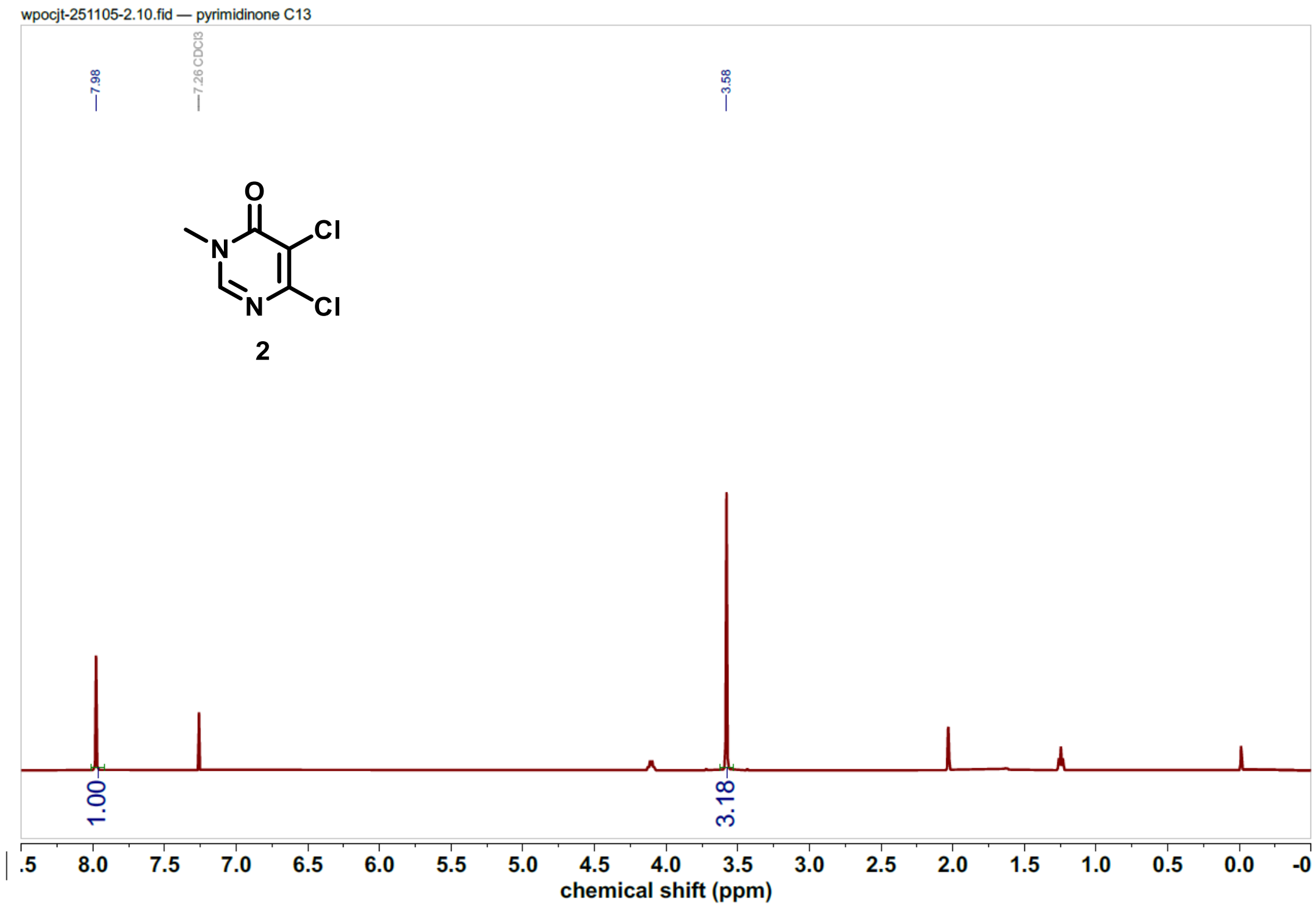

2, ^{13}C NMR (126 MHz, $CDCl_3$)

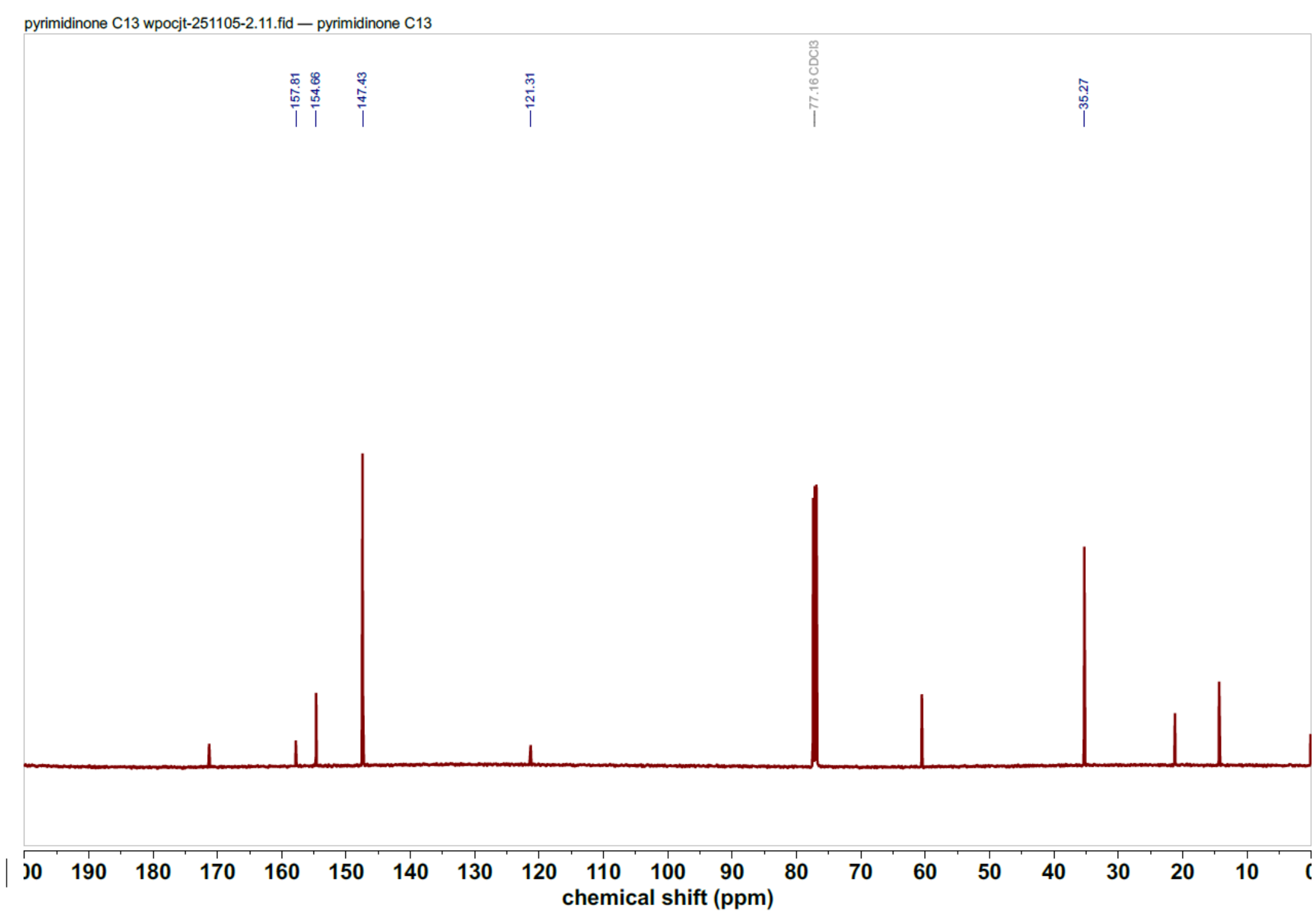

BZ2, ^{1}H NMR (500 MHz, methanol-d_4)

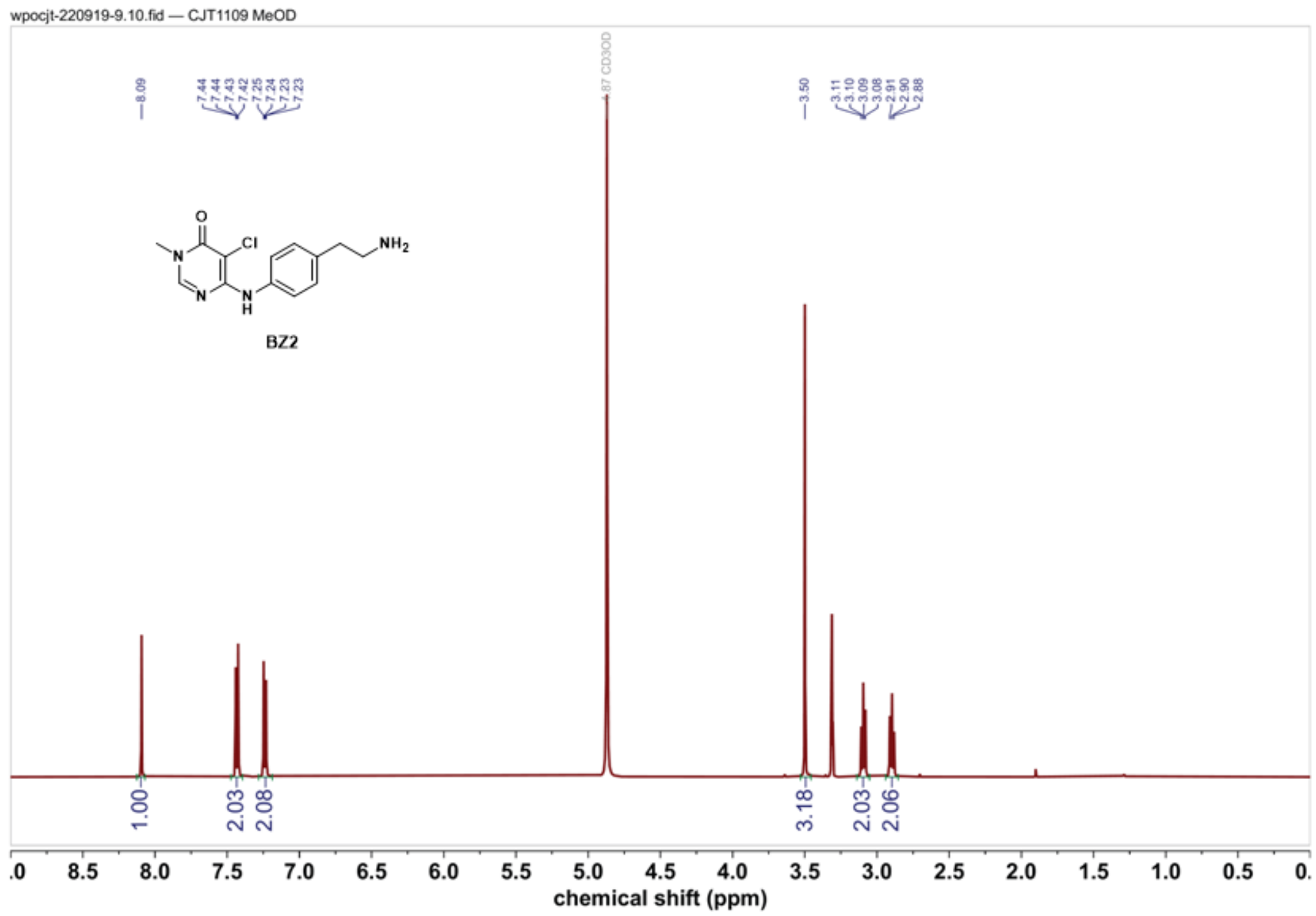

BZ2, ^{13}C NMR (500 MHz, methanol-d_4)

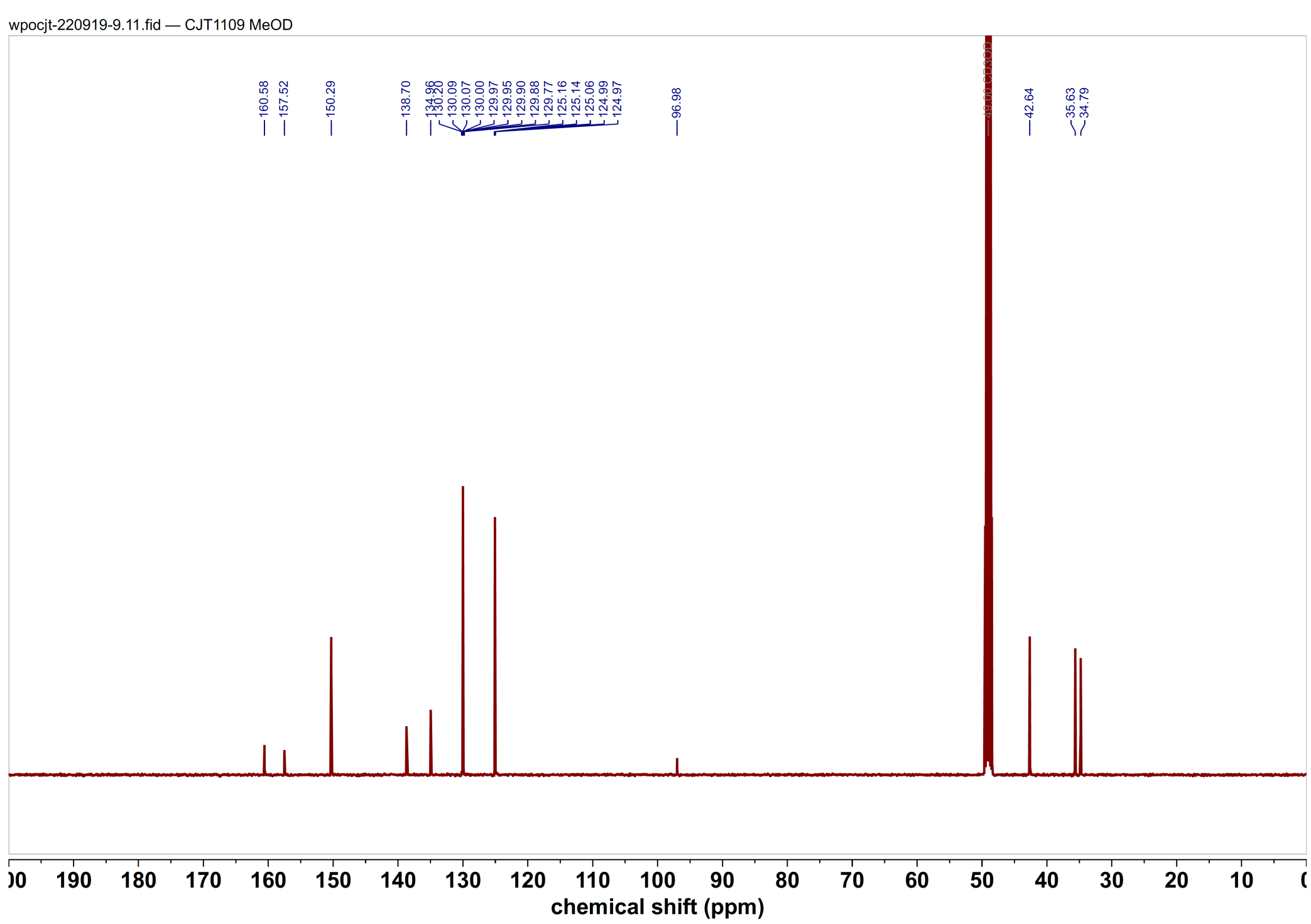

BZ2-neg, ^{1}H NMR (500 MHz, methanol-d_4)

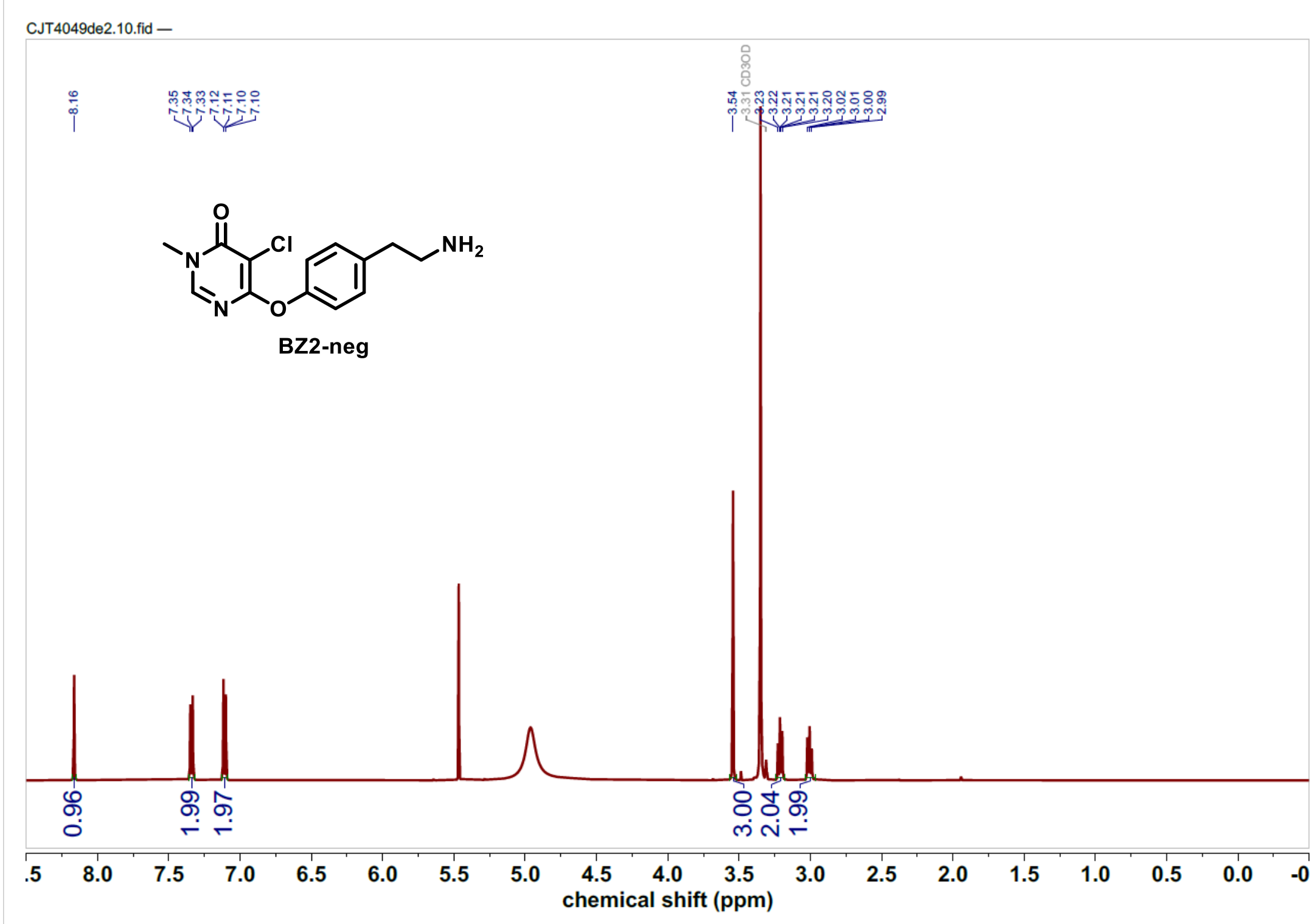

BZ2-neg, ^{13}C NMR (500 MHz, methanol-d_4)

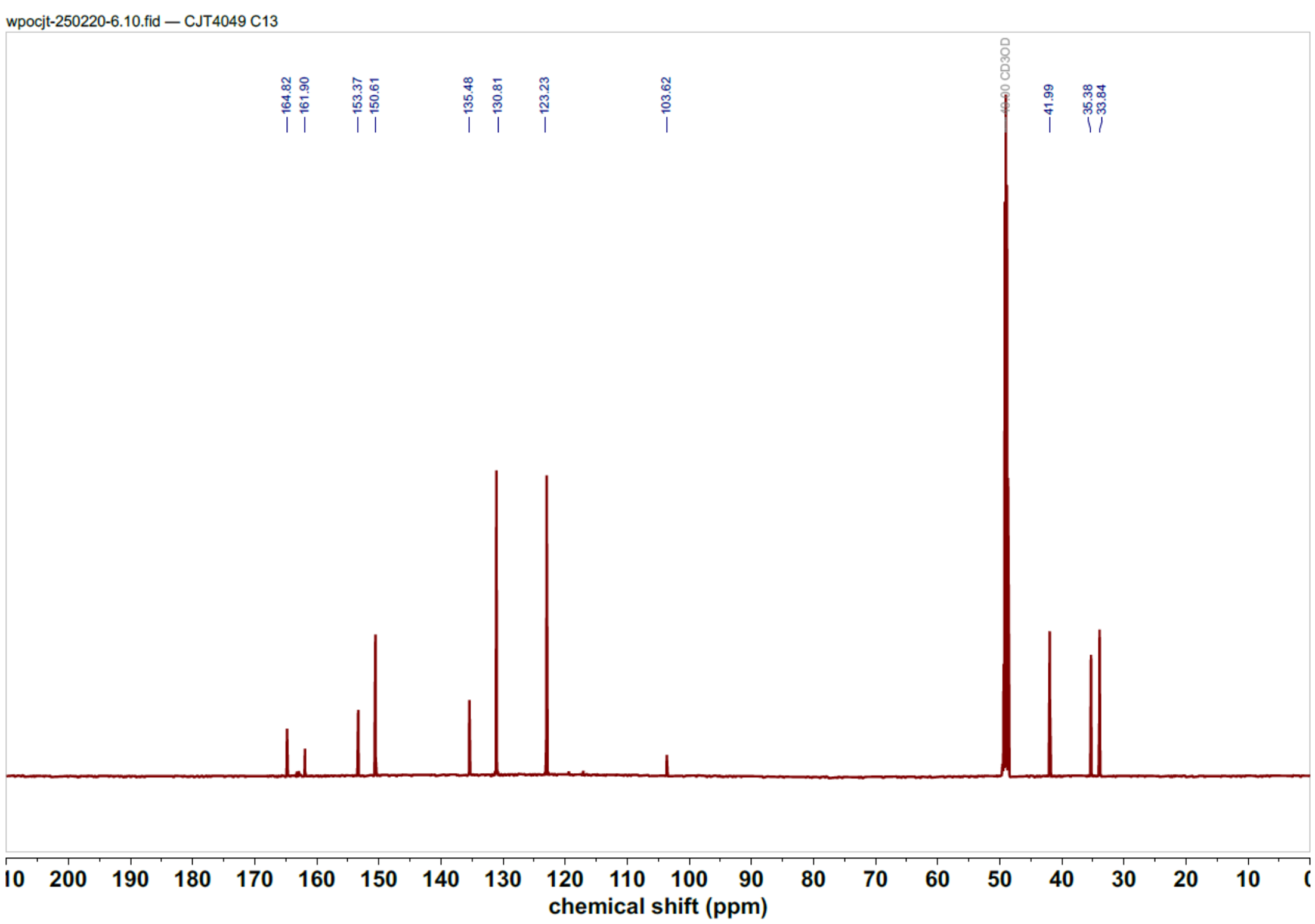

Protein purification and crystallization

Overexpression and purification of BRDs of BPTF and BRD9 were adapted from our previous studies[13,14] BPTF was dialysed against 50 mM HEPES pH 7.8, 100 mM NaCl, 1 mM DTT and concentrated to 16 mg/ml. Thin, needle-shaped BPTF crystals grew in the condition of 0.2 M NaCl and 23% PEG 3350 after 14-16 days. Optimized BPTF crystals were obtained by microseeding and were soaked in solution containing 1 mM BZ2 for 1 hour at room temperature (RT). Crystals were cryo-protected using the well solution supplemented with additional 10% glycerol. Co-crystallization of BPTF-BZ1 was achieved by incubating 2 mM BZ1 with BPTF for 1 hr at RT followed by hanging drop crystallization in a buffer of 0.1 M Tris-HCl pH 8.5, 0.2 M lithium sulfate and 24 % PEG 3350. BPTF-BZ1 crystals were cryoprotected with 35 % PEG 3350. Apo-BRD9 crystals were grown by the hanging drop method with the buffer containing 0.1 M Bis-Tris pH 6.5, 0.15 M ammonium sulfate and 29 % PEG 3350 and these crystals were used as seeds to obtain co-crystals of BRD9 with BZ1 and BZ2. 32 mg/ml of BRD9 was incubated with 3 mM of BZ1 and BZ2 compounds for 1 hour at RT. Co-crystals of BRD9 were grown in the same condition as apo BRD9 at 18˚C. 10% DMSO along with crystal conditions was used as a cryoprotectant.

X-ray diffraction data collection and structure refinement

X-ray diffraction data of BPTF-**BZ1**, BRD9-**BZ1** and BRD9-**BZ2** were collected at 100 K on beamline NSLS-II-AMX 17ID. BPTF-**BZ2** datasets were collected at 100 K on beamline APS SER-CAT 22ID. The diffraction images of BPTF-**BZ1** and BPTF-**BZ2** were indexed, integrated, and scaled using DIALS[15] and HKL2000,[16] respectively. Phases were obtained by rigid body refinement using 7M2E as the initial model. BRD9-**BZ1** and BRD9-**BZ2** datasets were processed by DIALS[15] using 5I7X as molecular replacement model. All the models were further refined and built using Phenix[17] and Coot,[18] respectively. Alternative conformations for amino acids were identified by using multi-conformer modeling tool *FLEXR*.[19,20] BPTF-**BZ1** and BRD9-**BZ1** crystal structures contain two copies in the asymmetric unit. Chain A for BPTF-**BZ1** and chain B for BRD9-**BZ1** were used for analysis as they were less impacted by crystal contacts and showed clearer ligand electron density. PyMOL (Schrödinger, New York) was used to render images and to detect hydrogen bonds. The 'align' function in PyMOL was used with default settings and BPTF-BZ2 as the reference structure to superpose structures shown in Figure 2. To investigate the B-factors and occupancies of ligands and waters in deposited structures, we re-refined them with Phenix using: `phenix.refine strategy=occupancies+individual_adp`. Normalized B-factors for stacking waters were calculated according to $(B_i - \langle B \rangle)/\sigma(B)$, where B_i is the B-factor of a water, $\langle B \rangle$ is the mean B-factor of all waters in that structure, and σ(B) is the standard deviation.

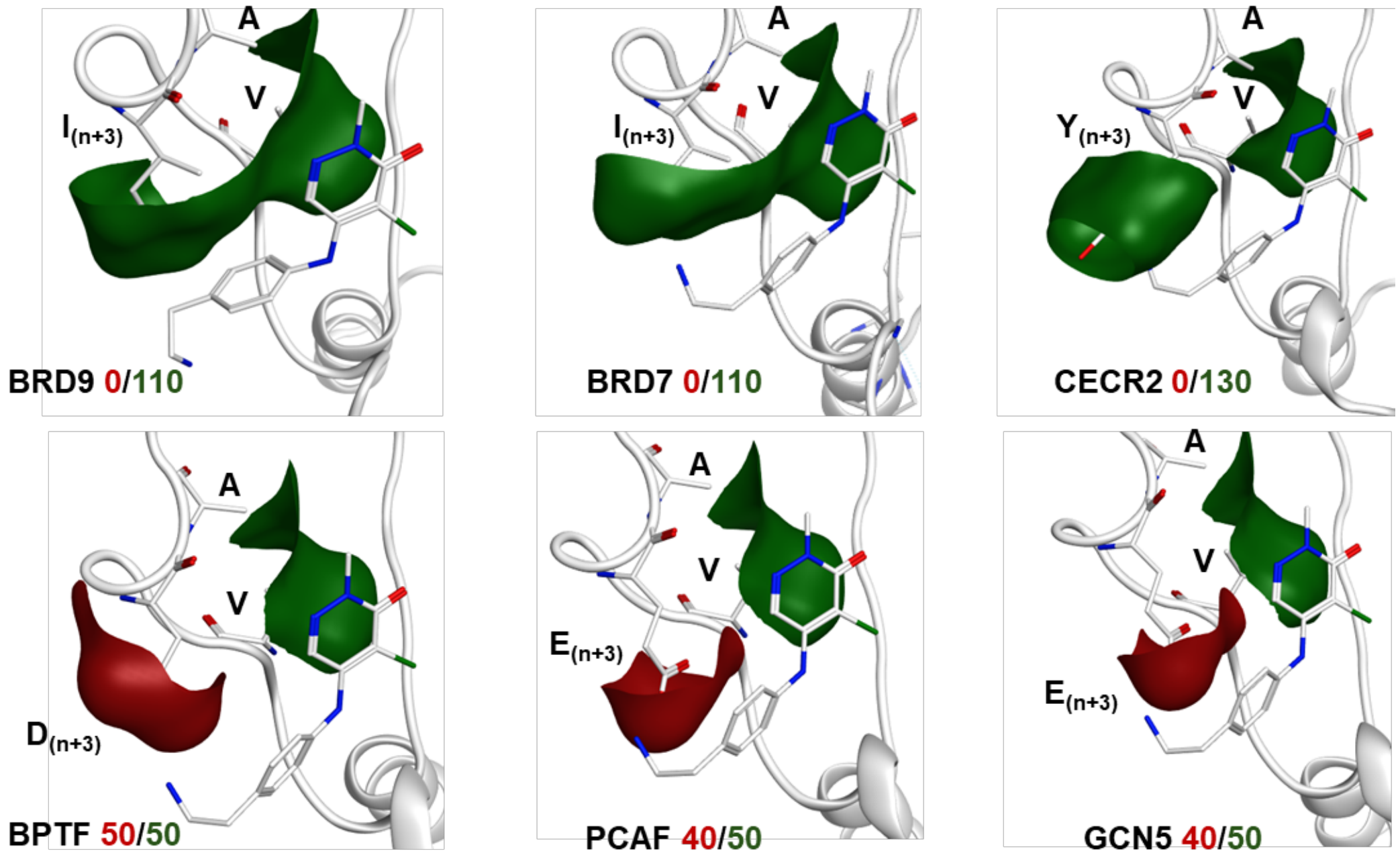

Figure S1. Quantification of polar (red) and lipophilic (green) surface areas in the vicinity of BZ1 docked to BPTF and off-targets. The presence of a trio of spatially adjacent Val, Ile, and Ala residues in BRD9 and BRD7 creates a 110 Å^2 continuous hydrophobic surface area in both proteins. A similar lipophilic environment is found in CECR2 and spans 130 Å^2, although this hydrophobic surface is fragmented and dependent on the conformation of the n+3 Tyr residue. In contrast, the corresponding regions in BPTF, PCAF, and GCN5 are approximately equally split between a lipophilic region centered around the N1 nitrogen in **BZ1** and a polar region adjacent to C6 which is bookended by the Asp or Glu at n+3. BZ1 was modeled using molecular docking. Values are reported in Å^2.

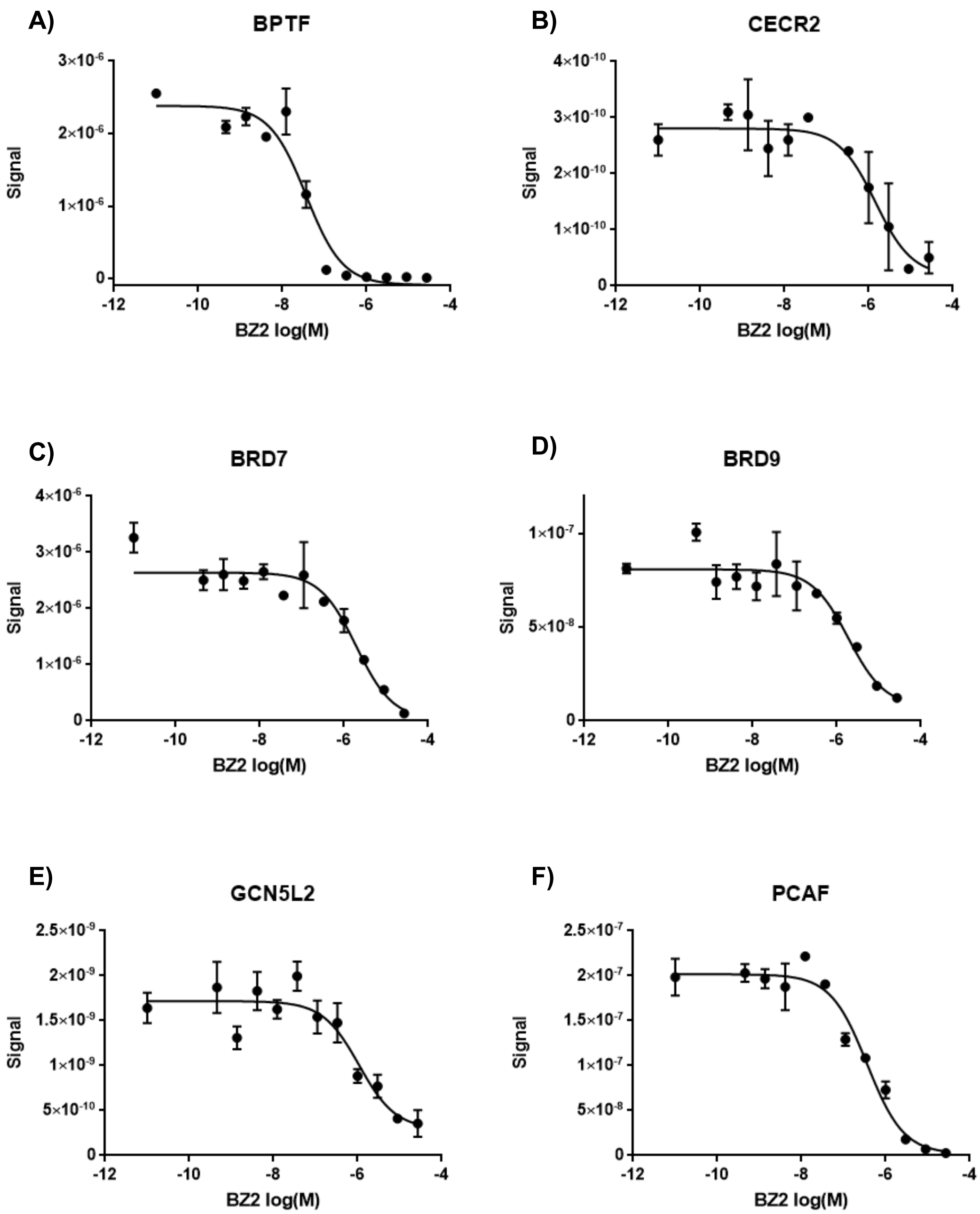

Figure S2. DiscoverX BROMOscan titrations of **BZ2** with (A) BPTF, (B) CECR2, (C) BRD7, (D) BRD9, (E) GCN5L2, and (F) PCAF bromodomains.

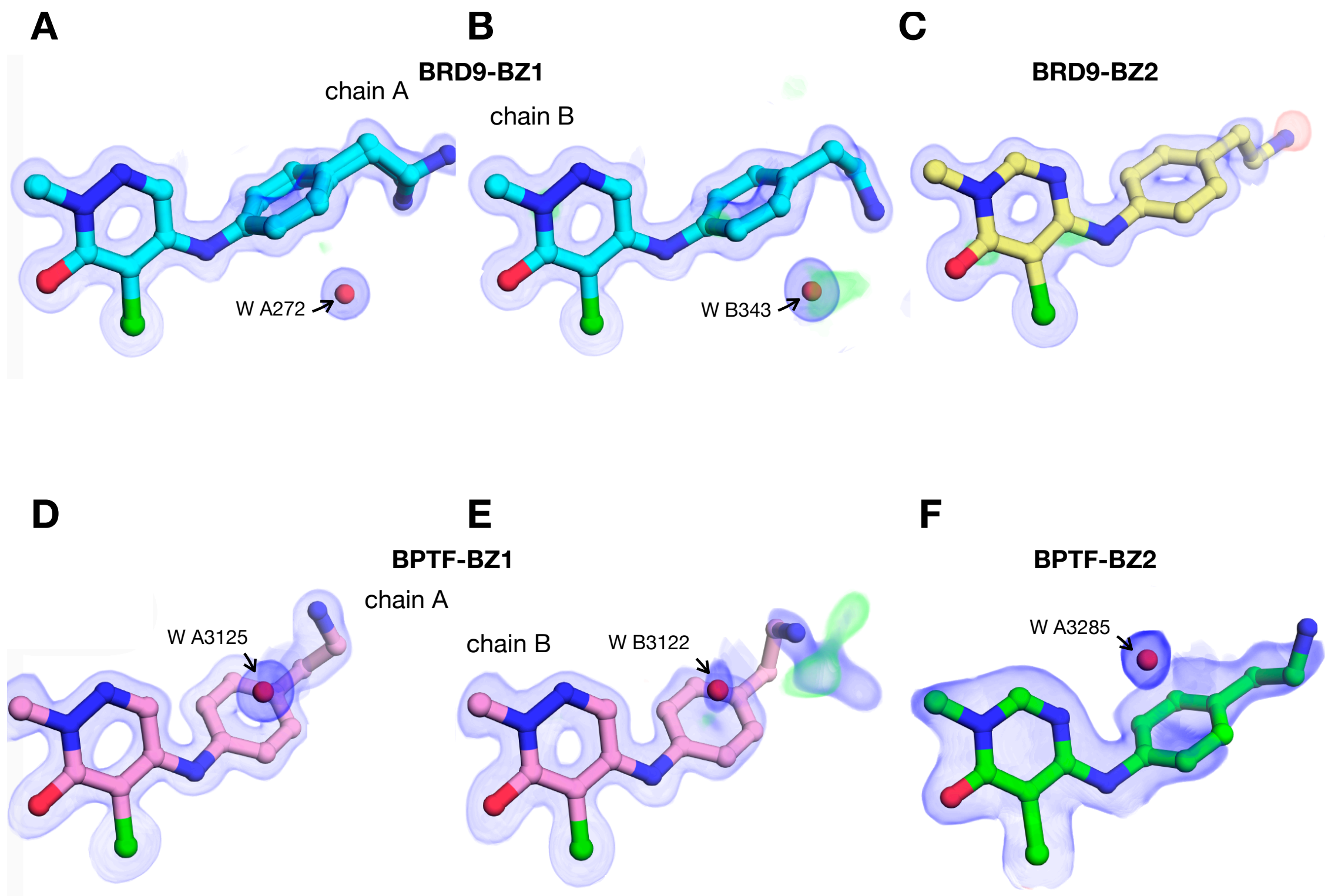

Figure S3. Electron density for ligand and stacking waters: (A and B) BPTF-BZ1, (C) BPTF-BZ2, (D and E) BRD9-BZ1, and (F) BRD9-BZ2. 2Fo-Fc maps are rendered in blue at 1σ and Fo-Fc maps are show in green at 3σ and red at -3σ.

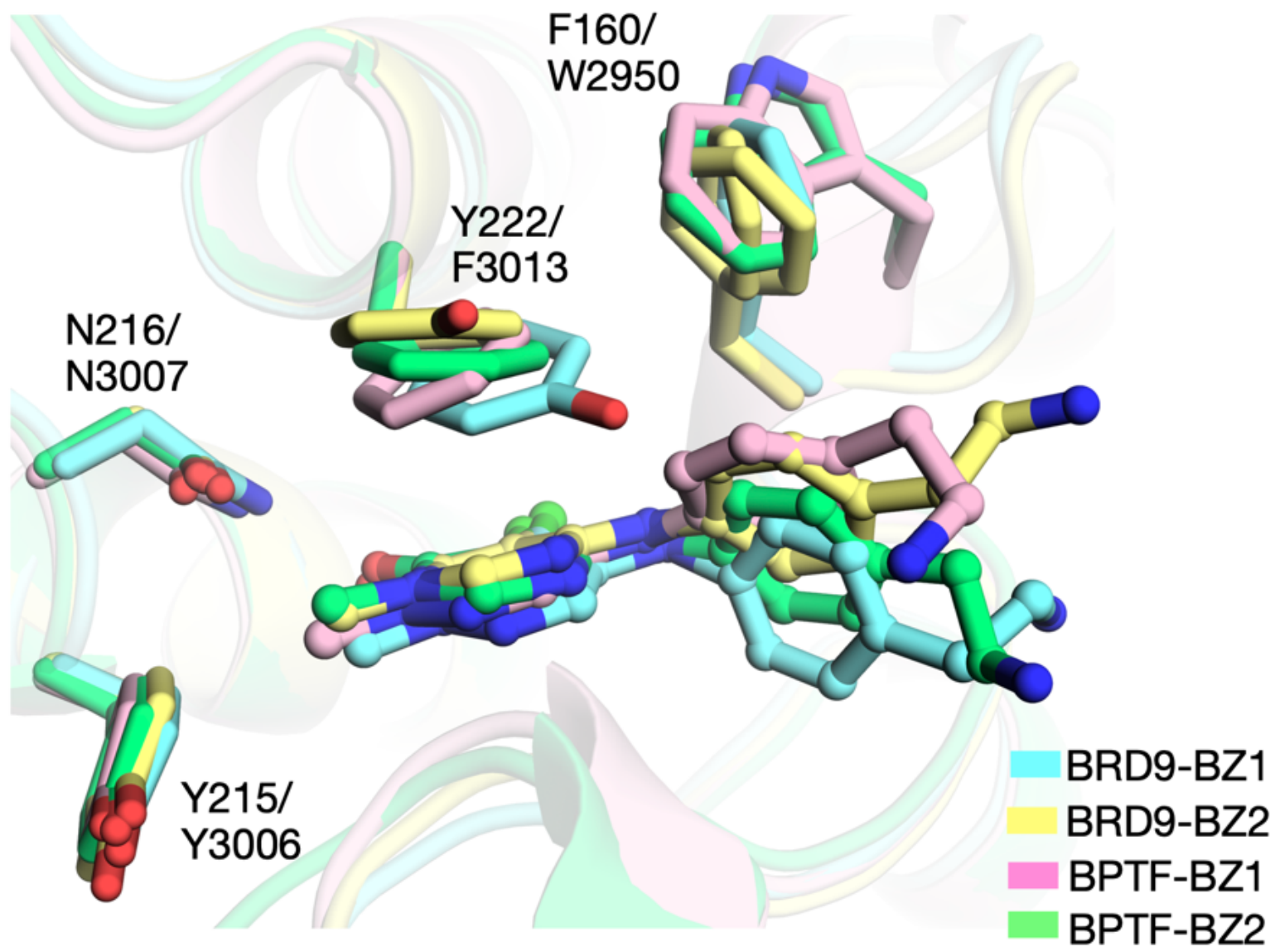

Figure S4. Structural superposition of BPTF and BRD9 bound BZ1 and BZ2. The superimposition of all four crystal structures highlights changes in the orientation of aromatic ring and amine tail of BZ1 and BZ2 in the binding pockets while the pyridazinone remains relatively anchored. Residue labels refer to BRD9/ BPTF residue numbering.

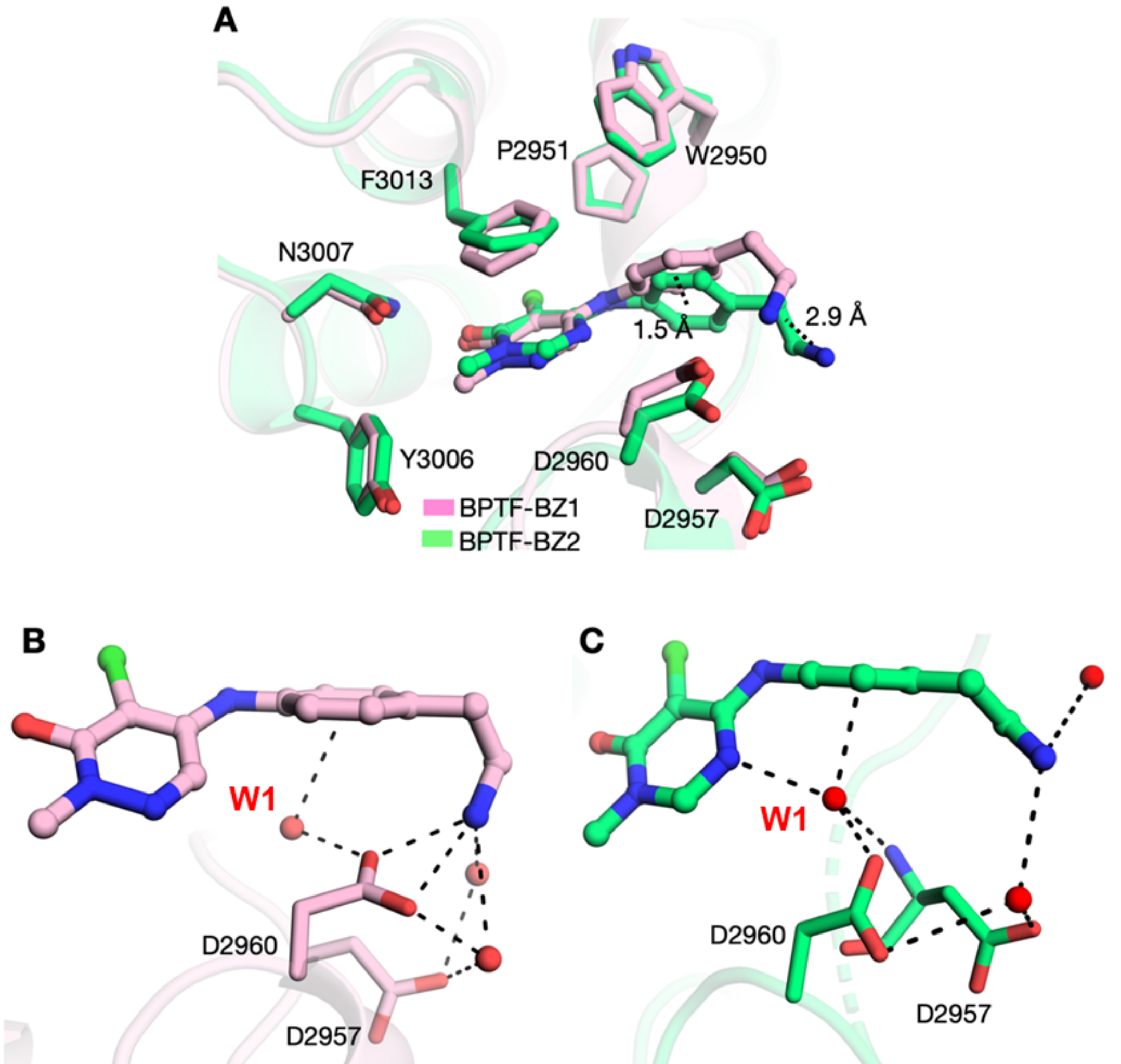

Figure S5. Comparison of BZ1 and BZ2 bound BPTF structures. (A) Superposition of BPTF-BZ1 and BPTF-BZ2. Dashed lines indicate distance between rings or amine tails. Direct or water-mediated interactions of aromatic ring and amine tails of (B) BZ1 and (C) BZ2 with BPTF. Dashed lines are interactions within 4 Å.

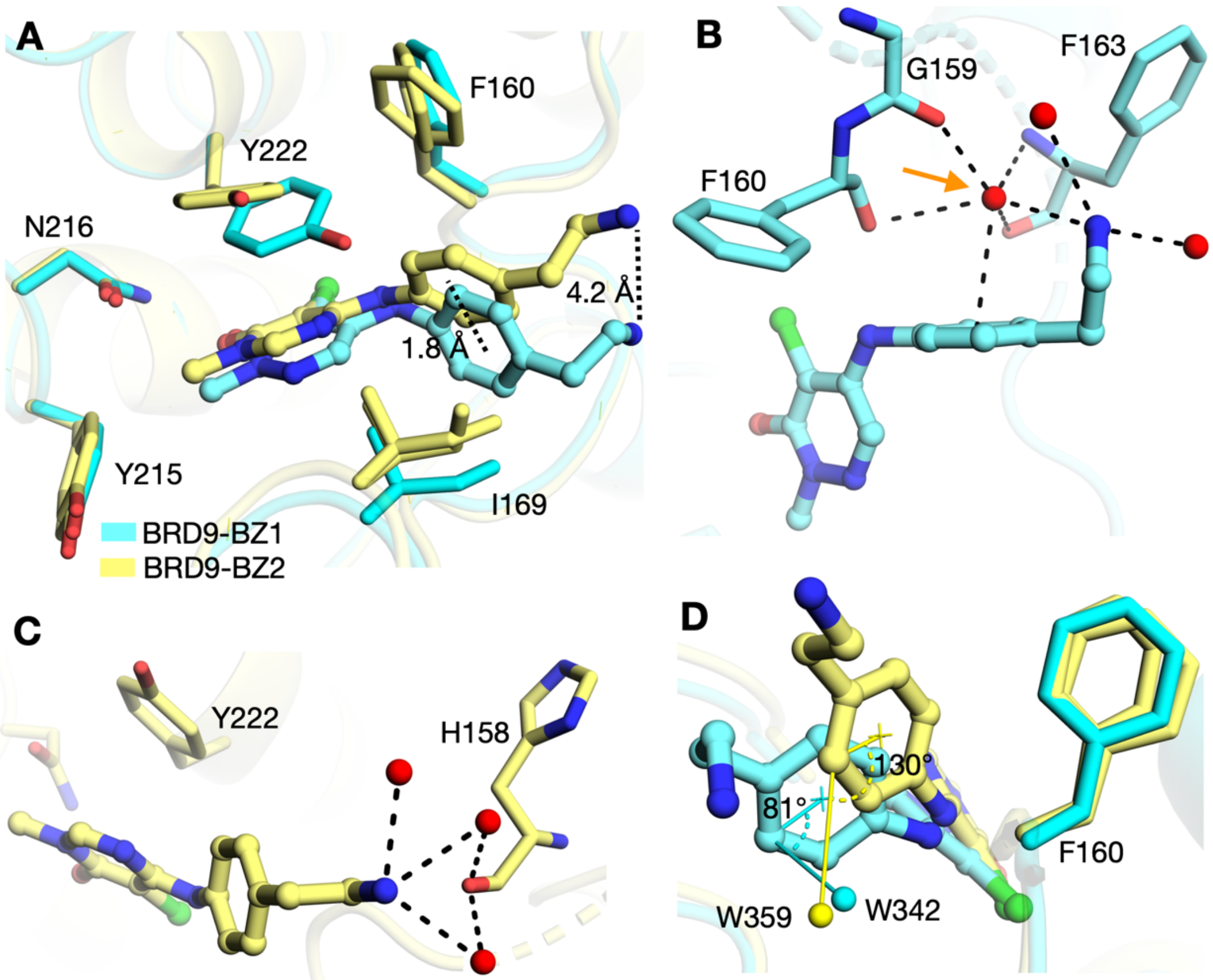

Figure S6. Comparison of BRD9-BZ1 and BRD9-BZ2 structures. (A) Structural superposition of BZ1 and BZ2 bound to BRD9. Dashed lines indicate distance between rings or amine tails. Direct or water-mediated interactions (dashed lines) of aromatic ring and amine tails of BZ1 (B) and BZ2 (C) with BRD9 (interactions are within 3.5 Å); orange arrow indicates a pi-stacking water. (D) The figure shows the placement of BZ2's aromatic ring away from the water. Angle (in degrees) of water in BZ2 bound BRD9 structure is increased and 'out of plane' compared to the water in BRD9-BZ1 structure.

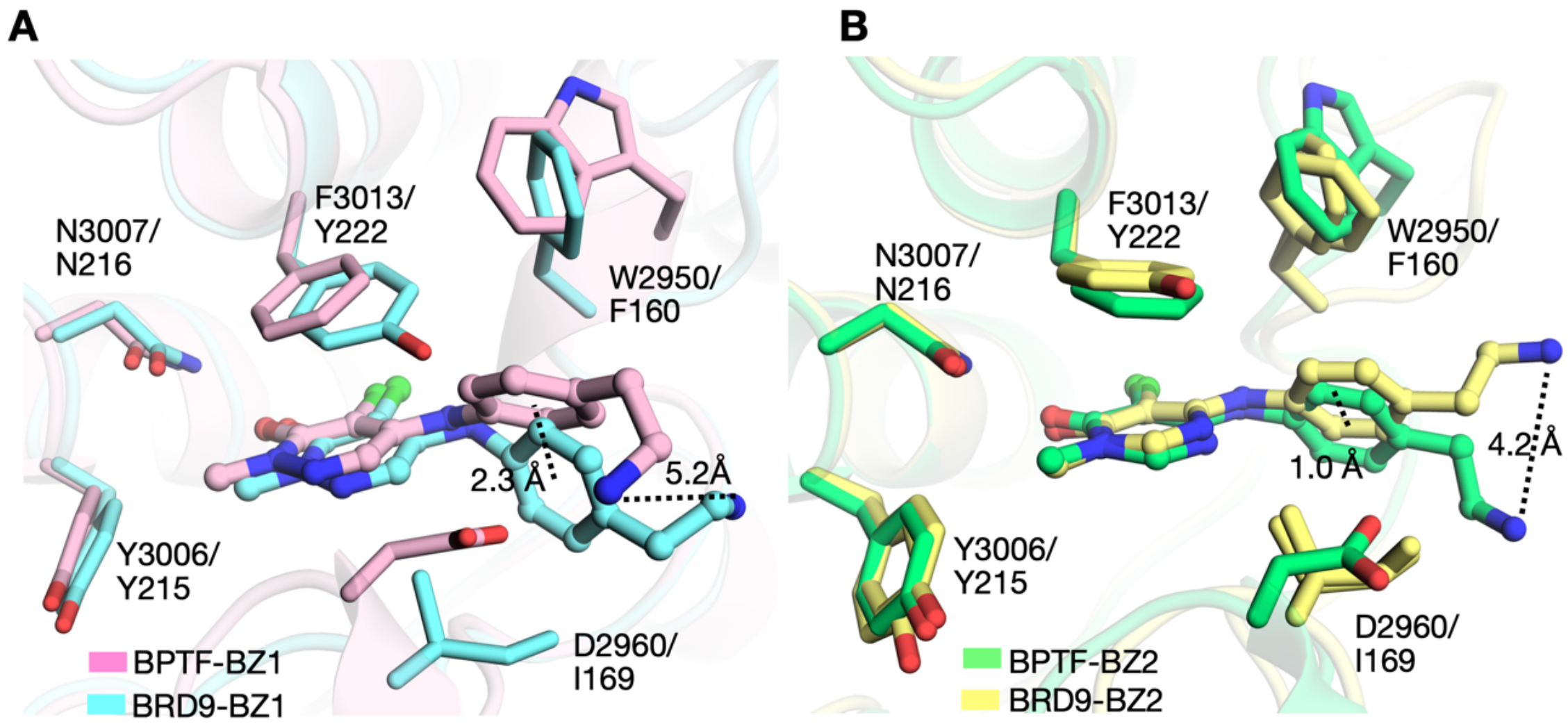

Figure S7. Comparison of BZ1 in the context of BPTF vs BRD9, and BZ2 in the context of BPTF vs BRD9. (A) Superposition of BPTF-BZ1 and BRD9-BZ1 crystal structures. (B) Superposition of BPTF-BZ2 and BRD9-BZ2 structures. Dashed lines indicate a difference in the placement of BZ1 or BZ2 between BPTF and BRD9 structures.

Table S1. The Asp_n-x-x-Asp_{n+3} sequence pattern is unique to BPTF.

Target	residue (n)	residue (n+1)	residue (n+2)	residue (n+3)
**BPTF**	**Asp**	Pro	Asn	**Asp**
**PCAF**	**Lys**	Arg	Thr	**Glu**
**GCN5**	**Lys**	Lys	Ser	**Glu**
**CECR2**	**Asp**	Glu	Ser	**Tyr**
**BRD7**	**Thr**	Asp	Phe	**Ile**
**BRD9**	**Thr**	Asp	Ala	**Ile**

General Procedure for the AlphaScreen Assay

His_9-tagged BPTF and BRD4 D1 were expressed and purified as described previously.[13] The AlphaScreen assay procedures for the BPTF and BRD4 bromodomains were adapted from the manufacturer's protocol (PerkinElmer). Nickel chelate (Ni-NTA) acceptor beads and streptavidin donor beads were purchased from PerkinElmer (catalogue no. 6760619M). The biotinylated Histone H4 KAc5,8,12,16 peptide was purchased from EpiCypher with the following sequence: Ac-SGRGK(Ac)GGK(Ac)GLGK(Ac)GGAK(Ac)RHRKVLR-Peg(Biot).

All reagents were diluted in a buffer consisting of 50 mM HEPES, 100 mM NaCl, 0.1% BSA, and 0.05% CHAPS, pH 7.4. Serial dilutions were prepared with varying concentrations of small molecules and fixed His-tagged protein concentration (see table) in 0.25% DMSO. 5 µL of these solutions were added to a 384-well white plate, followed by 5 µL biotinylated H4 KAc5,8,12,16 (Cat. # 12-0034, EpiCypher), to a final concentration of 100 nM. 10 µL of premixed AlphaScreen streptavidin donor and nickel chelate acceptor beads (6760619M, PerkinElmer) were added to a final concentration of 20 µg/mL and incubated for 30 minutes at room temperature. The plate was then measured on a Tecan Spark reader, measuring 520-620 nM. Data was plotted in GraphPad Prism 9 using a sigmoidal 4-parameter logistic (4PL) curve fit.

Protein	Source	[Protein] nM	[Peptide] nM
BPTF	Expressed and purified as described previously	30	100
BRD9	Cat. # RD-11-214, Reaction Biology	60	100
CECR2	Cat. # RD-11-210, Reaction Biology	60	100
BRD4 D1	Expressed and purified as described previously	15	50

Table S2. Binding affinities of BZ1 and BZ2 to BPTF and other BRD-containing proteins from the AlphaScreen and TR-FRET competition assays. Values were averaged across two technical replicates (N = 3).

Target	Assay	BZ1 (nM)	BZ1 Selectivity	BZ2 (nM)	BZ2 Selectivity	BZ1/BZ2
**BPTF**	AlphaScreen	70 (± 10)	1.0	220 (± 20)	1.0	3.1
**CECR2**	AlphaScreen	450 (± 30)	6.4	1400 (± 100)	6.4	3.1
**BRD9**	AlphaScreen	43 (± 10)	0.6	2800 (± 500)	12.7	65.1
**BRD4-D1**	AlphaScreen	31,000	442.9	NB*	-	-
**BRD4-D1**	TR-FRET			Not active	-	-

*NB = no binding at top conc. 2.5 μM

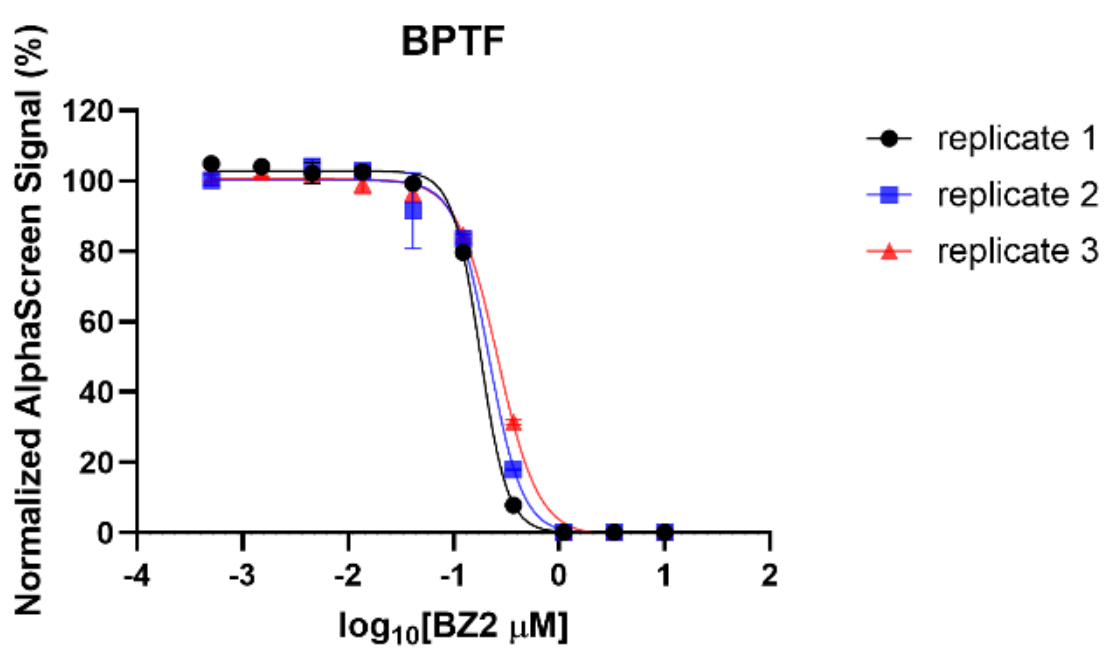

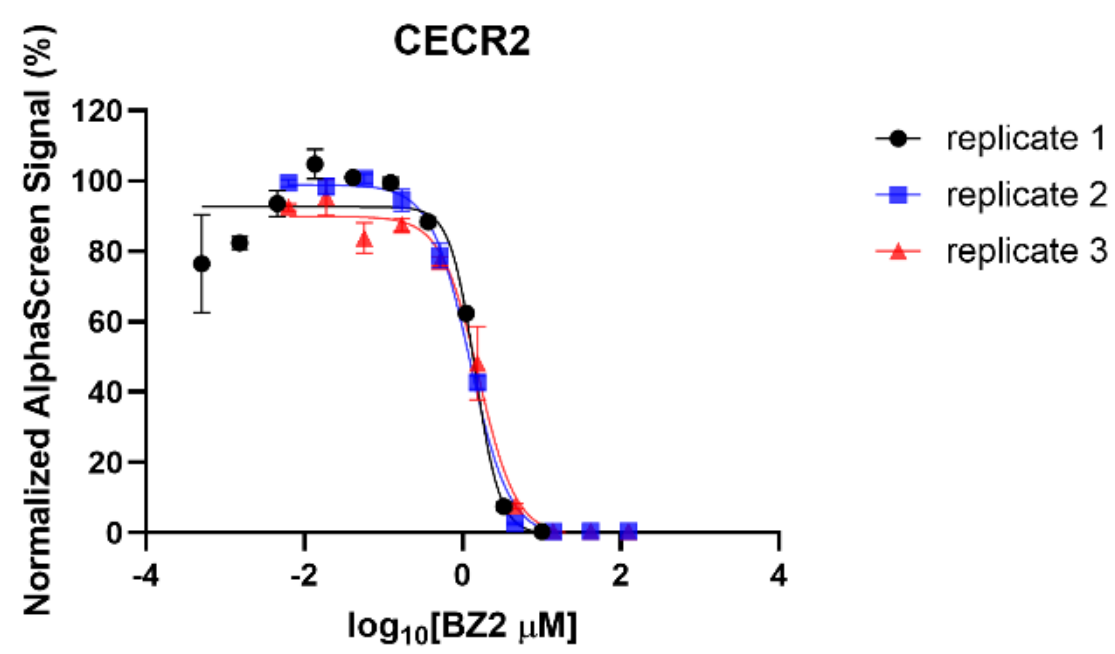

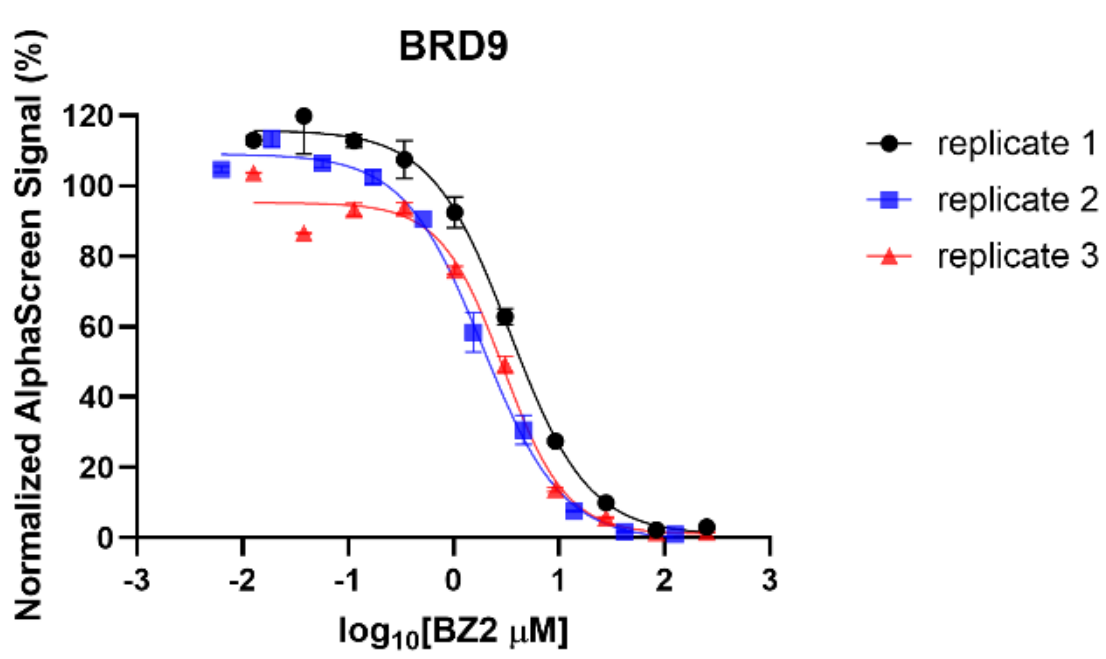

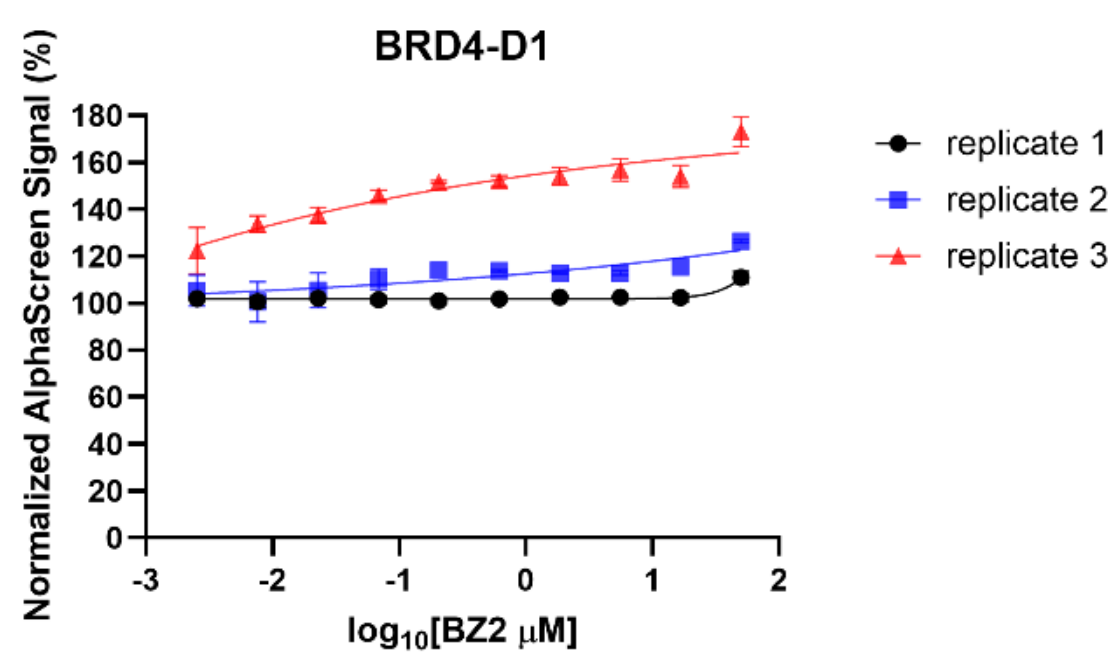

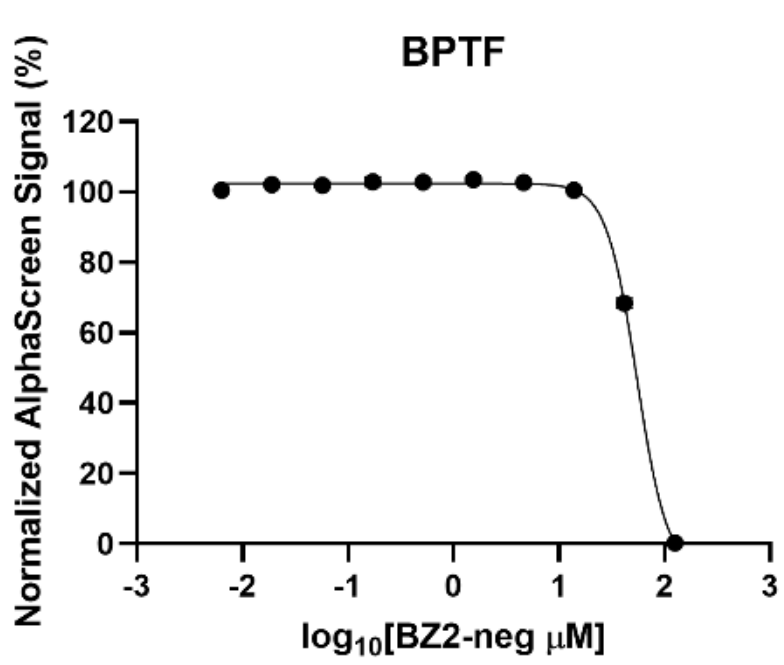

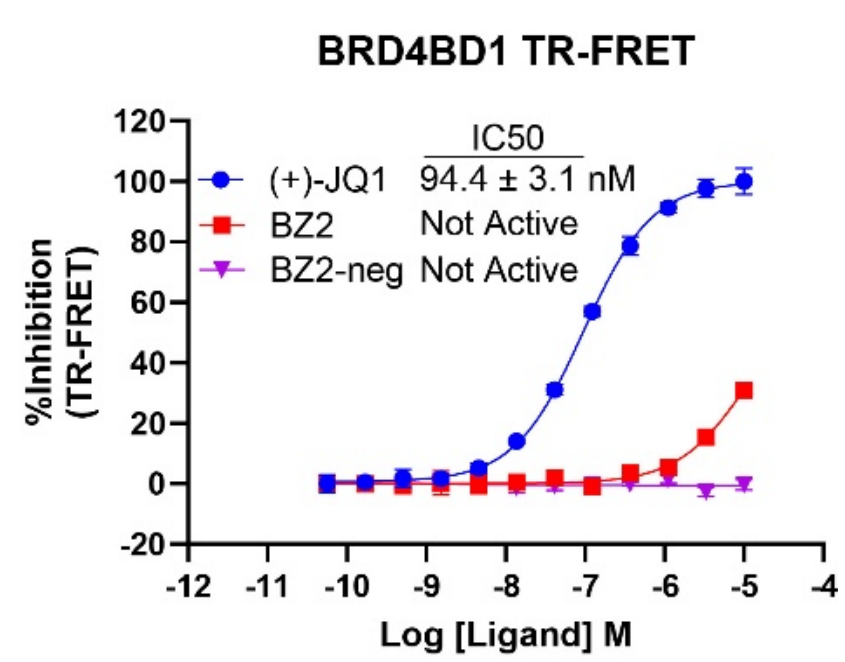

Table S3. Crystallographic data processing and refinement statistics. Values in parentheses are from high resolution bins.

Parameter	BPTF-BZ1	BPTF-BZ2	BRD9-BZ1	BRD9-BZ2
PDB ID	9ZUK	8F6G	9ZU3	9ZU4
Wavelength	0.92	0.97	0.92	0.92
Resolution range (Å)	26.31 - 1.349 (1.38 - 1.35)	33.43 - 1.947 (2.23 - 1.95)	37.43 - 1.15 (1.18 - 1.15)	36.08 - 1.05 (1.08 - 1.05)
Space group	P 1	P 1 21 1	P 2 21 21	P 21 21 21
Unit cell	a= 27.144, b= 38.452, c= 57.22, α= 81.54, β= 76.19, γ= 87.69	a= 26.976, b= 66.859 c= 39.564, α= 90, β= 104.94, γ= 90	a= 35.761, b= 46.275, c= 127.275, α= 90, β= 90, γ= 90	a= 40.307, b= 47.55, c= 55.377, α= 90, β= 90, γ= 90
Unique reflections	47067 (3231)	8537 (2507)	74315 (4717)	49431 (3343)
Multiplicity	3.6 (3.6)	2.3 (1.9)	6.7 (6.9)	13.1 (10.8)
Completeness (%)	96.30 (93.30)	85.58 (76.02)	97.83 (94.95)	98.12 (93.72)
Mean I/sigma(I)	12.7 (1.1)	7.77 (1.12)	15.9 (1.9)	26.7 (3.7)
Wilson B-factor	14.32	22.46	11.14	10.4
R-merge	0.076 (0.735)	0.105 (0.609)	0.059 (0.645)	0.048 (0.313)
R-meas	0.089 (0.861)	0.133 (0.793)	0.064 (0.698)	0.050 (0.328)
R-pim	0.046 (0.445)	0.081 (0.502)	0.024 (0.264)	0.014 (0.098)
CC1/2	0.997 (0.706)	0.989 (0.399)	0.998 (0.666)	0.999 (0.968)
Reflections used in refinement	47067 (3231)	8537 (2507)	74315 (4717)	49431 (3343)
Reflections used for R-free	1945 (141)	392 (105)	2057 (136)	1963 (135)
R-work	0.1523 (0.2676)	0.1918 (0.2270)	0.1309 (0.1817)	0.1256 (0.1333)
R-free	0.1920 (0.3567)	0.2290 (0.2876)	0.1553 (0.2140)	0.1448 (0.1775)
Number of non-hydrogen atoms	2377	1164	2300	1163
macromolecules	2045	1025	1829	969
ligands	38	19	72	19
solvent	294	120	399	175
Protein residues	230	121	208	103
RMS(bonds)	0.007	0.004	0.010	0.008
RMS(angles)	0.91	0.71	1.00	0.97
Ramachandran favored (%)	100	100	99.51	100
Ramachandran allowed (%)	0	0	0.49	0
Ramachandran outliers (%)	0	0	0	0
Rotamer outliers (%)	0.45	0.89	0.51	0.98
Clash score	5.43	2.46	1.61	1.05
Average B-factor	21.05	27.81	16.30	13.93
macromolecules	19.85	27.37	14.04	12.44

Table S4. Pi-stacking water stability based on normalized B-factor.

Model	PDB ID	Space group	Resolution (Å)	Ligand occupancy*	Water ID	Water occupancy*	Normalized Water B-factor
BPTF-Cmpd10	7RWP	$P22_12_1$	1.73	0.78	A3262	0.98	-0.98
BPTF-Cmpd11	7RWN	$P12_11$	1.39	0.87	A3296	0.98	-1.01
BPTF-Cmpd12	7RWQ	$P22_12_1$	1.9	0.96	A3172	0.89	-0.66
BPTF-Cmpd13	7RWO	$P12_11$	1.58	0.91	A3162	1.0	-0.73
BPTF-Cmpd19	7M2E	$P12_11$	1.75	0.94	A3517	1.0	-0.71
BPTF-BZ1	9ZUK	P1	1.35	0.83	A3125	0.76	-1.67
BPTF-BZ2	8F6G	$P12_11$	1.95	0.92	A3285	0.7	-2.24
BRD9-BZ1	9ZU3	$P22_12_1$	1.15	0.88	B343	0.88	-1.16
BRD9-BZ2	9ZU4	$P2_12_12_1$	1.05	0.90	NA	NA	NA
BRD9-Ligand 14	6YQR	P1	1.68	0.88	A401	0.87	-1.73

*Occupancies and B-factors were re-refined for previously deposited structures.

Table S5. Cell permeability studies of BZ2.

Assay	Readout	Value
Caco-2 Permeability	AVG Papp A/B (nm/s)	11.66
Caco-2 Permeability	SD Papp A/B	2.74
Caco-2 Permeability	AVG Papp B/A (nm/s)	14.54
Caco-2 Permeability	SD Papp B/A	10.95
Caco-2 Permeability	Efflux Ratio (B2A/A2B)	1.25
MDCKII Permeability	AVG Papp A/B (nm/s)	495.89
MDCKII Permeability	SD Papp A/B	163.51
MDCKII Permeability	AVG Papp B/A (nm/s)	11.68
MDCKII Permeability	SD Papp B/A	2.61
MDCKII Permeability	Efflux Ratio (B2A/A2B)	0.02
MDCKII (MDR1 OE) Permeability	AVG Papp A/B (nm/s)	113.84
MDCKII (MDR1 OE) Permeability	SD Papp A/B	12.83
MDCKII (MDR1 OE) Permeability	AVG Papp B/A (nm/s)	23.85
MDCKII (MDR1 OE) Permeability	SD Papp B/A	11.27
MDCKII (MDR1 OE) Permeability	Efflux Ratio (B2A/A2B)	0.21

Protocols based on: Nishiguchi G, et al. *J Med Chem.* **2021** Jun 10;64(11):7296-7311 for the MDCKII assays and Rakesh, Bruhn D et al. *Bioorg Med Chem.* **2012** Oct 15;20(20):6063-72. For the Caco-2 assays

Chemistry for TR-FRET probes. (+)-JQ1 carboxylic acid and BODIPY FL EDA HCl were purchased from MedChemExpress USA (Monmouth Junction, NJ).

BODIPY FL-(+)-JQ1 (**Scheme S1**). (+)-JQ1 carboxylic acid (40 mg, 100 µmole), BODIPY FL EDA HCl (37 mg, 100 µmole), EDAC (23.1 mg, 120 µmole), DMAP (14.7 mg, 120 µmole) and DIPEA (26.3 µL, 19.5 mg, 150 µmole) were solubilized in DCM (10 mL). The mixture was stirred under room temperature for 60 min. By then, LC-MS monitoring demonstrated the starting material (+)-JQ1 carboxylic acid was depleted, and the desired compound **BODIPY FL-(+)-JQ1** was formed. The reaction mixture was quenched by 20 mL brine. The organic DCM layer was separated. The aqueous solution was extracted by DCM (10 mL x 3). All DCM solutions were combined and washed with brine (10 mL x 3). The DCM solution was dried by anhydrous Na_2SO_4 and solvent was

removed under reduced pressure. The residue was purified by the reversed-phase flash chromatography to yield the product **BODIPY FL-(+)-JQ1** (37.1 mg, purity: 97.1%, yield: 51.8%). ^{1}H NMR (500 MHz, DMSO-d_6) δ 8.25 (t, J = 5.5 Hz, 1H), 7.95 (t, J = 5.1 Hz, 1H), 7.69 (s, 1H), 7.48 (d, J = 8.3 Hz, 2H), 7.42 (d, J = 8.2 Hz, 2H), 7.09 (d, J = 4.0 Hz, 1H), 6.35 (d, J = 4.1 Hz, 1H), 6.30 (s, 1H), 4.51 (t, J = 7.1 Hz, 1H), 3.26 – 3.21 (m, 2H), 3.19 – 3.16 (m, 4H), 3.08 (t, J = 7.8 Hz, 2H), 2.59 (s, 3H), 2.49 – 2.48 (m, 2H), 2.47 (s, 3H), 2.40 (s, 3H), 2.26 (s, 3H). ^{13}C NMR (126 MHz, DMSO-d_6) δ 169.21, 167.98, 161.28, 157.38, 156.03, 153.38, 148.09, 142.33, 134.98, 133.46, 132.69, 131.22, 130.50, 128.93, 128.40, 128.08, 127.82, 127.17, 126.72, 123.61, 118.50, 114.84, 52.03, 36.72, 36.57, 35.89, 32.04, 22.16, 12.75, 12.29, 10.92, 9.54, 9.24. ESI-TOF HRMS *m/z*: [M + H]$^+$ Calcd for $C_{35}H_{37}BClF_2N_8O_2S^+$ 717.2504; Found 717.2537.

Scheme S1. Synthesis of BODIPY FL-(+)-JQ1

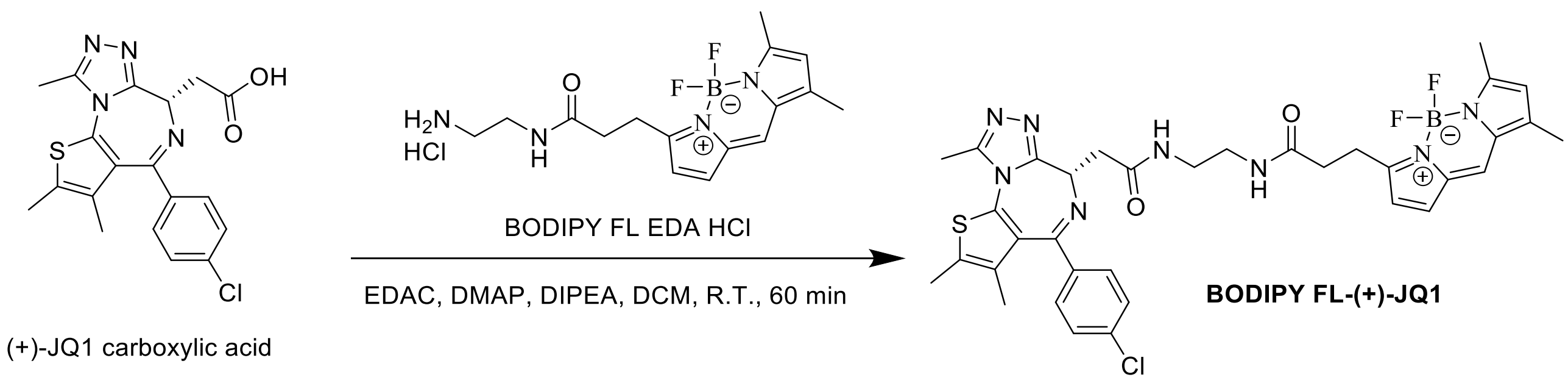

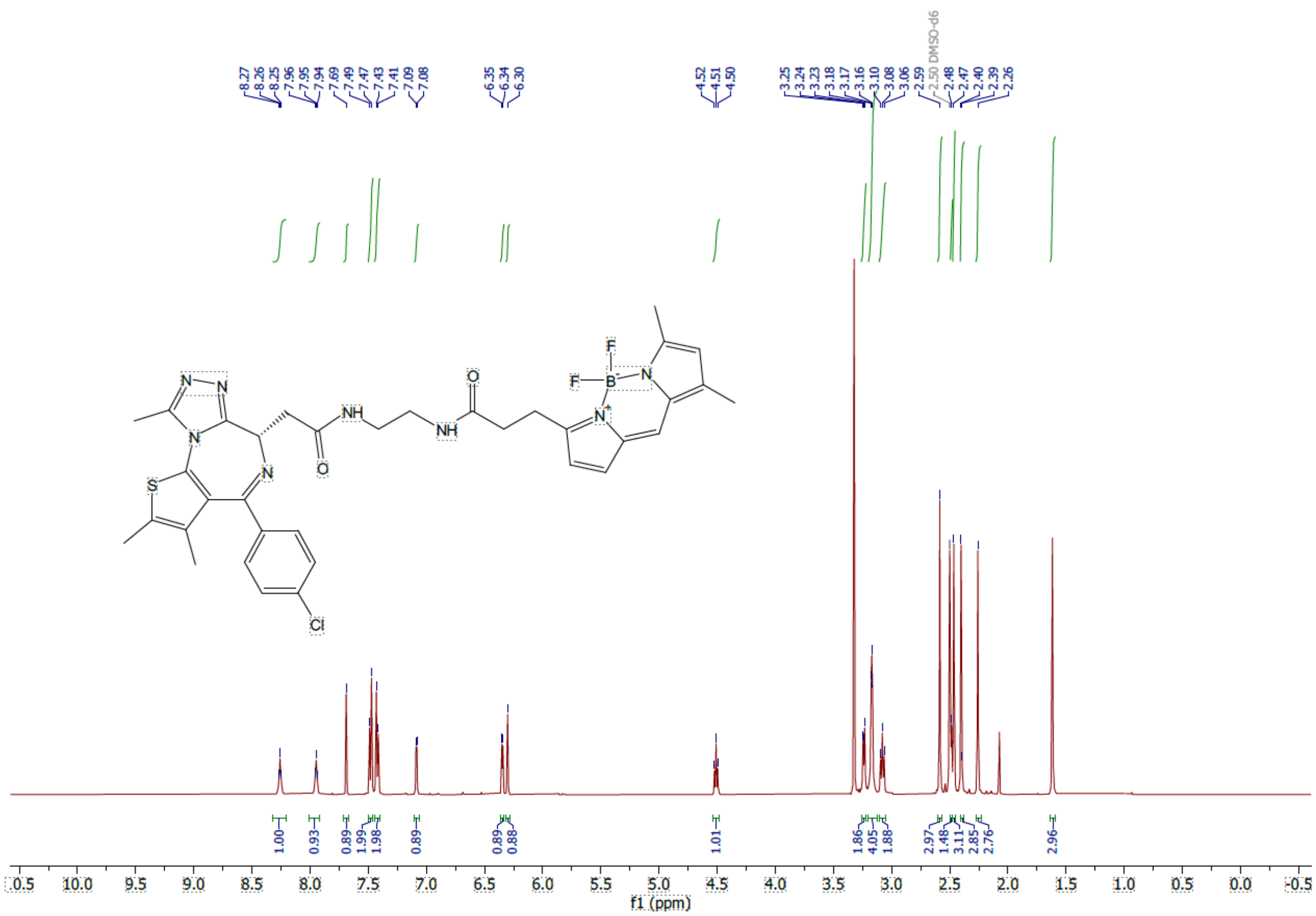

Figure S8. ^{1}H NMR spectrum of BODIPY FL-(+)-JQ1.

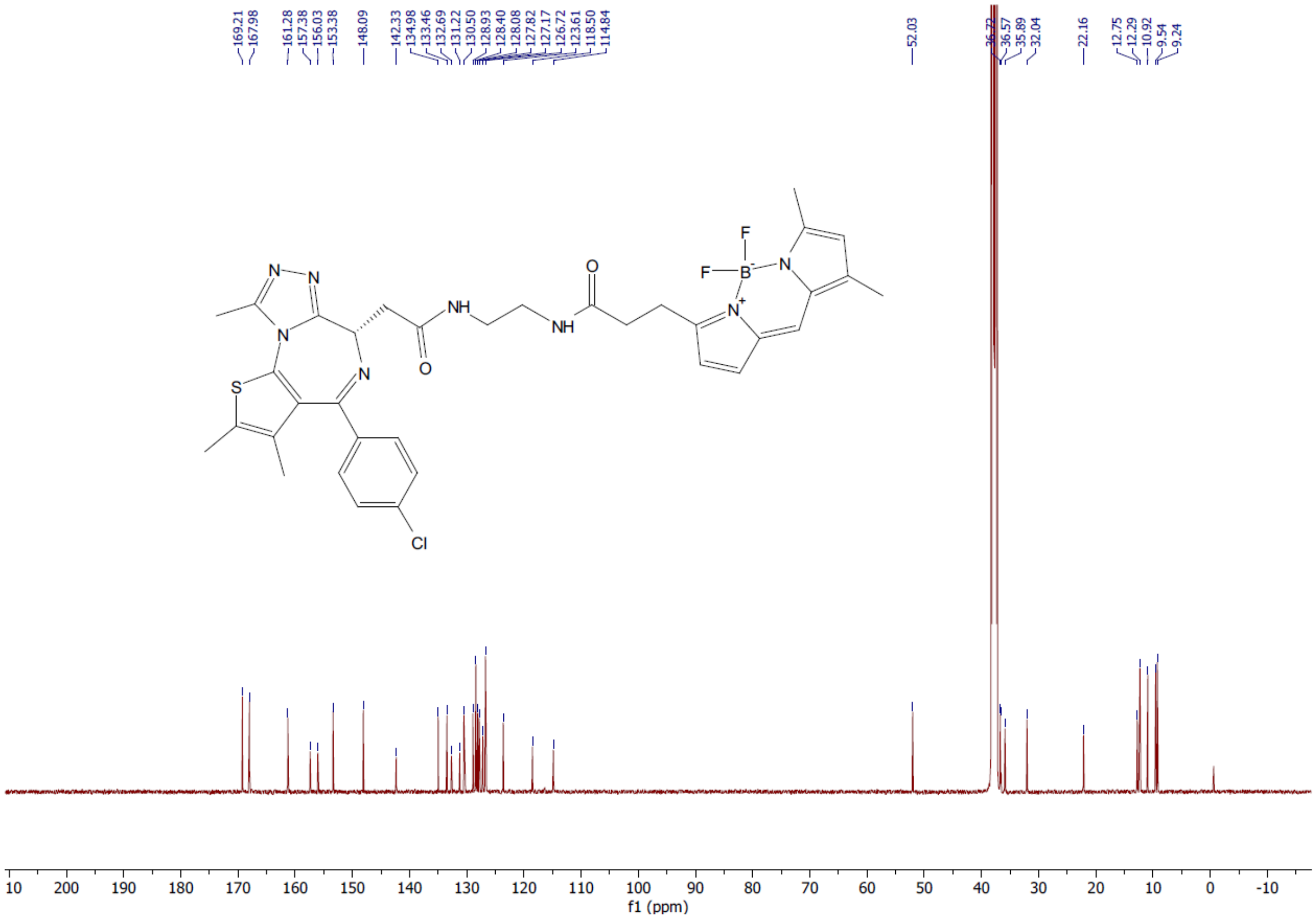

Figure S9. ^{13}C NMR spectrum of BODIPY FL-(+)-JQ1.

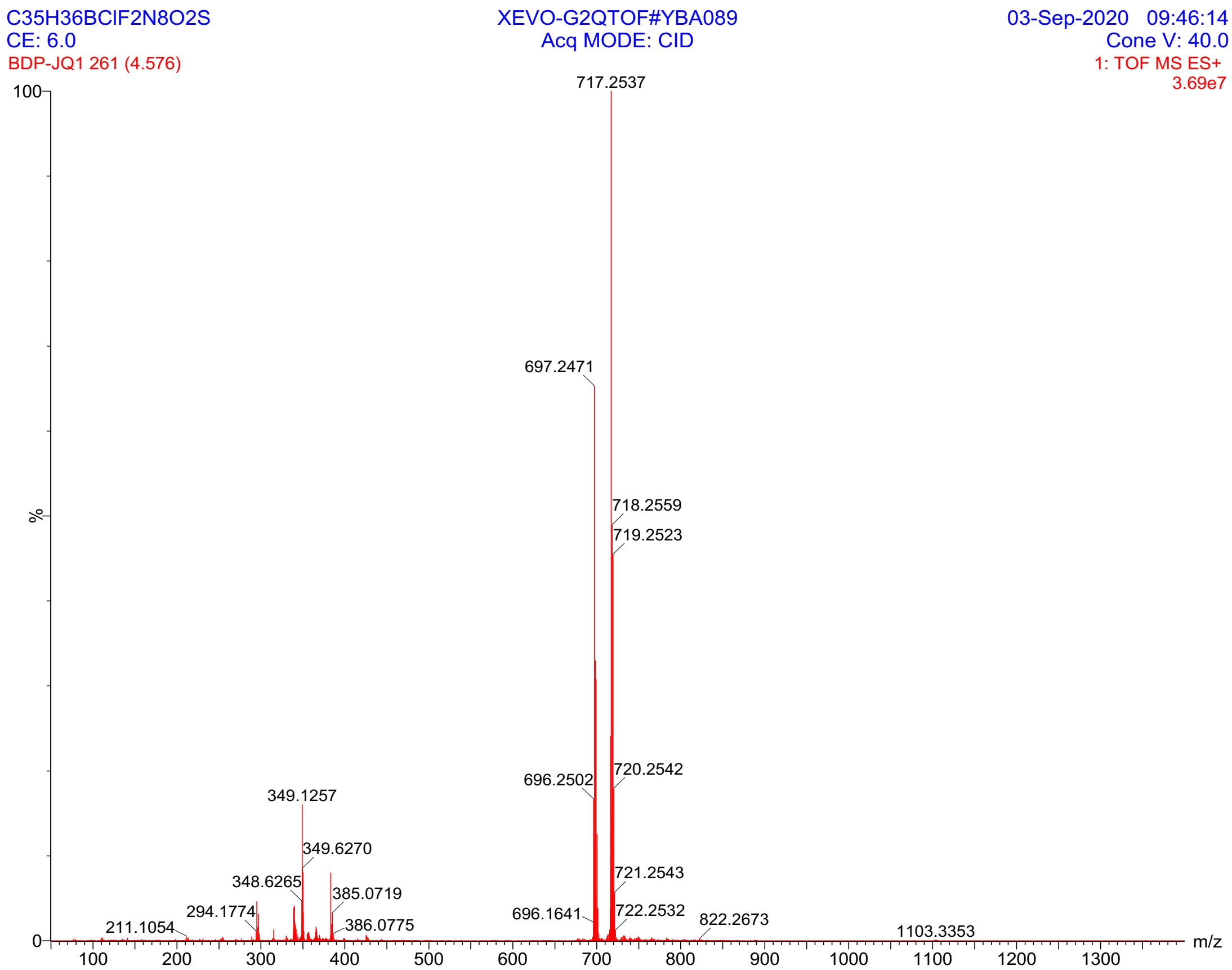

Figure S10. HRMS spectrum of BODIPY FL-(+)-JQ1.

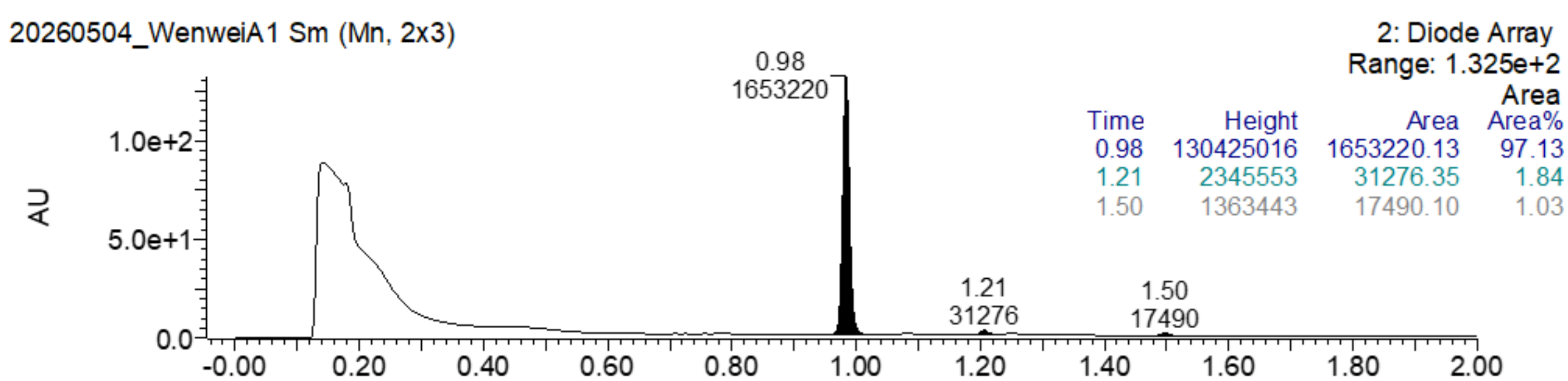

Figure S11. HPLC purity chromatogram of BODIPY FL-(+)-JQ1.

Biology. GST-BRD4BD1 was purchased from BPS Bioscience (San Diego, CA). (+)-JQ1 was purchased from Cayman Chemical (Ann Arbor, MI). Tb-anti-GST, 1 M Tris pH 7.5, Triton X-100,

BSA and DTT were purchased from Thermo Fisher Scientific Inc. (Waltham, MA). Distilled water was purchased from Research Products International (Mount Prospect, IL) and used for assay buffer preparation. 384-well Echo LDV plates were purchased from Beckman Coulter, Inc. (Indianapolis, IN). Black 384-well low volume assay plates and 384-well white tissue culture-treated plates were purchased from Revvity (Waltham, MA).

TR-FRET ligand dose response inhibition with BODIPY FL-(+)-JQ1 as the probe against GST-BRD4BD1 protein

In a black 384-well low volume assay plate, 5 µL per well of 15.6 nM BODIPY FL-(+)-JQ1 in the TR-FRET assay buffer with 0.2% DMSO was first dispensed. After spun down at 1,000 rpm for 30 seconds in the Eppendorf centrifuge, the plate was dispensed with 10 nL per well of DMSO, 10 mM (+)-JQ DMSO stock, or 1-to-3 dilutions of selected ligand DMSO stock solutions in an Echo LDV 384-well plate by the Echo 655T Acoustic Liquid Handler. A mixture of Tb-anti-GST (4 nM) and GST-BRD4 (4 nM) in 5 µL TR-FRET assay buffer was then dispensed. The plate was shaken on the microplate shaker to ensure assay components in each well mixed thoroughly and then spun down at 1,000 rpm for 30 seconds in the Eppendorf centrifuge. The final BODIPY FL-(+)-JQ1 concentration was 7.8 nM (K_d concentration). The final DMSO concentration was 0.2% in all assay wells with 0.1% from the BODIPY FL-(+)-JQ1 and the other 0.1% from testing ligands. The final concentration was: 0.2% DMSO (negative control), 10 µM (+)-JQ1 (positive control), or ranged from 56.5 pM-to-10 µM in a 1-to-3 dilution pattern for 12 concentration levels when ligands were tested in a dose dependent manner. The TR-FRET signal for each well was determined with the PHERAstar *FS* plate reader by measuring the fluorescence emission ratio of 10,000 × 520 nm/490 nm, using a 337-nm excitation filter, a 100-µs delay, and a 200-µs integration time at 90-minute incubation time.

In the presence of 7.8 nM BODIPY FL-(+)-JQ1, the group with Tb-anti-GST (2 nM), GST-BRD4BD1 (2 nM) and (+)-JQ (10 µM) was served as the positive control; the group with Tb-anti-GST (2 nM), GST-BRD4 (2 nM) and DMSO was served as the negative control. The %inhibition of each ligand at a specific concentration was calculated by Equation S1:

$$\%\text{Inhibition} = 100\% - 100\% \times \left(\frac{\text{Signal}_{\text{Ligand}} - \text{Signal}_{10\mu\text{M (+)-JQ1}}}{\text{Signal}_{\text{DMSO}} - \text{Signal}_{10\mu\text{M (+)-JQ1}}}\right) \qquad \text{Equation S1}$$

The calculated %inhibition values for each ligand at various concentrations were fit with the Sigmoidal dose-response fitting equation in the GraphPad Prism software to derive an IC_{50} value for the ligand, when applicable.

Statistics

All experiments were tested in quadruplicate for three times. Results are expressed as the mean ± standard deviation (SD) from the three experiments.

References:

[1] D. A. Case, H. M. Aktulga, K. Belfon, D. S. Cerutti, G. A. Cisneros, V. W. D. Cruzeiro, N. Forouzesh, T. J. Giese, A. W. Götz, H. Gohlke, S. Izadi, K. Kasavajhala, M. C. Kaymak, E. King, T. Kurtzman, T.-S. Lee, P. Li, J. Liu, T. Luchko, R. Luo, M. Manathunga, M. R. Machado, H. M. Nguyen, K. A. O'Hearn, A. V. Onufriev, F. Pan, S. Pantano, R. Qi, A. Rahnamoun, A. Risheh, S. Schott-Verdugo, A. Shajan, J. Swails, J. Wang, H. Wei, X. Wu, Y. Wu, S. Zhang, S. Zhao, Q. Zhu, T. E. Cheatham 3rd, D. R. Roe, A. Roitberg, C. Simmerling, D. M. York, M. C. Nagan, K. M. Merz Jr, *J. Chem. Inf. Model.* **2023**, *63*, 6183–6191.

[2] D. A. Case, D. S. Cerutti, V. W. D. Cruzeiro, T. A. Darden, R. E. Duke, M. Ghazimirsaeed, G. M. Giambaşu, T. J. Giese, A. W. Götz, J. A. Harris, K. Kasavajhala, T.-S. Lee, Z. Li, C. Lin, J. Liu, Y. Miao, R. Salomon-Ferrrer, J. Shen, R. Snyder, J. Swails, R. C. Walker, J. Wang, X. Wu, J. Zeng, T. E. Cheatham Iii, D. R. Roe, A. Roitberg, C. Simmerling, D. M. York, M. C. Nagan, K. M. Merz Jr, *J. Chem. Inf. Model.* **2025**, *65*, 7835–7843.

[3] X. He, V. H. Man, W. Yang, T.-S. Lee, J. Wang, *J. Chem. Phys.* **2020**, *153*, 114502.

[4] A. Jakalian, D. B. Jack, C. I. Bayly, *J. Comput. Chem.* **2002**, *23*, 1623–1641.

[5] J. A. Maier, C. Martinez, K. Kasavajhala, L. Wickstrom, K. E. Hauser, C. Simmerling, *J. Chem. Theory Comput.* **2015**, *11*, 3696–3713.

[6] W. L. Jorgensen, J. Chandrasekhar, J. D. Madura, R. W. Impey, M. L. Klein, *J. Chem. Phys.* **1983**, *79*, 926–935.

[7] E. Neria, S. Fischer, M. Karplus, *J. Chem. Phys.* **1996**, *105*, 1902–1921.

[8] C. W. Hopkins, S. Le Grand, R. C. Walker, A. E. Roitberg, *J. Chem. Theory Comput.* **2015**, *11*, 1864–1874.

[9] T. Darden, D. York, L. Pedersen, *The Journal of Chemical Physics* **1993**, *98*, 10089–10092.

[10] B. R. Miller 3rd, T. D. McGee Jr, J. M. Swails, N. Homeyer, H. Gohlke, A. E. Roitberg, *J. Chem. Theory Comput.* **2012**, *8*, 3314–3321.

[11] A. Onufriev, D. Bashford, D. A. Case, *Proteins* **2004**, *55*, 383–394.

[12] P. J. Slavish, L. Chi, M.-K. Yun, L. Tsurkan, N. E. Martinez, B. Jonchere, S. C. Chai, M. Connelly, M. B. Waddell, S. Das, G. Neale, Z. Li, W. R. Shadrick, R. R. Olsen, K. W. Freeman, J. A. Low, J. E. Price, B. M. Young, N. Bharatham, V. A. Boyd, J. Yang, R. E. Lee, M. Morfouace, M. F. Roussel, T. Chen, D. Savic, R. K. Guy, S. W. White, A. A. Shelat, P. M. Potter, *Cancer Res.* **2020**, *80*, 3507–3518.

[13] P. D. Ycas, H. Zahid, A. Chan, N. M. Olson, J. A. Johnson, S. K. Talluri, E. Schonbrunn, W. C. K. Pomerantz, *Org. Biomol. Chem.* **2020**, *18*, 5174–5182.

[14] H. Zahid, C. R. Buchholz, M. Singh, M. F. Ciccone, A. Chan, S. Nithianantham, K. Shi, H. Aihara, M. Fischer, E. Schönbrunn, C. O. dos Santos, J. W. Landry, W. C. K. Pomerantz, *J. Med. Chem.* **2021**, *64*, 13902–13917.

[15] G. Winter, D. G. Waterman, J. M. Parkhurst, A. S. Brewster, R. J. Gildea, M. Gerstel, L. Fuentes-Montero, M. Vollmar, T. Michels-Clark, I. D. Young, N. K. Sauter, G. Evans, *Acta Crystallogr. D Struct. Biol.* **2018**, *74*, 85–97.

[16] Z. Otwinowski, W. Minor, *Methods Enzymol.* **1997**, *276*, 307–326.

[17] D. Liebschner, P. V. Afonine, M. L. Baker, G. Bunkóczi, V. B. Chen, T. I. Croll, B. Hintze, L. W. Hung, S. Jain, A. J. McCoy, N. W. Moriarty, R. D. Oeffner, B. K. Poon, M. G. Prisant, R. J. Read, J. S. Richardson, D. C. Richardson, M. D. Sammito, O. V. Sobolev, D. H. Stockwell, T. C. Terwilliger, A. G. Urzhumtsev, L. L. Videau, C. J. Williams, P. D. Adams, *Acta Crystallogr. D Struct. Biol.* **2019**, *75*, 861–877.

[18] P. Emsley, B. Lohkamp, W. G. Scott, K. Cowtan, *Acta Crystallogr. D Biol. Crystallogr.* **2010**, *66*, 486–501.

[19] T. R. Stachowski, M. Fischer, *Acta Crystallogr. D Struct. Biol.* **2023**, *79*, 354–367.

[20] T. R. Stachowski, M. Fischer, *J. Appl. Crystallogr.* **2024**, *57*, 580–586.

Author Contributions:K.B. and S.N. performed X-ray crystallography; T.R.S. performed crystallographic refinement and comparative structural analysis; C.T. synthesized the compounds and conducted biophysical experiments; S.Z. performed MD simulations at 300 K; P.S. and S.D. performed MD simulations and molecular docking studies; R.S.K. contributed optimizing the NanoBRET assay M.S. synthesized the NanoBRET tracer and performed the NanoBRET assay; X.F. and LY performed and oversaw the cell permeability assays; L.F. and A.P. conducted neuroblastoma experiments; C.B. synthesized and characterized compounds 10 and 19; W.L., Y.L., and T.C. performed the TR-FRET studies; M.F., A.S., and W.C.K.P. oversaw the experiments and the interpretation of the data. C.T. and W.C.K.P. wrote the initial draft.
